# Interferon Regulatory Factor 9 Promotes Lung Cancer Progression via Regulation of Versican

**DOI:** 10.3390/cancers13020208

**Published:** 2021-01-08

**Authors:** David Brunn, Kati Turkowski, Stefan Günther, Andreas Weigert, Thomas Muley, Mark Kriegsmann, Hauke Winter, Reinhard H. Dammann, Georgios T. Stathopoulos, Michael Thomas, Andreas Guenther, Friedrich Grimminger, Soni S. Pullamsetti, Werner Seeger, Rajkumar Savai

**Affiliations:** 1 Max Planck Institute for Heart and Lung Research, Member of the German Center for Lung Research (DZL), Member of the Cardio-Pulmonary Institute (CPI), 61231 Bad Nauheim, Germany; david.brunn@mpi-bn.mpg.de (D.B.); kati.turkowski@mpi-bn.mpg.de (K.T.); stefan.guenther@mpi-bn.mpg.de (S.G.); soni.pullamsetti@mpi-bn.mpg.de (S.S.P.); werner.seeger@mpi-bn.mpg.de (W.S.); 2 Institute of Biochemistry I, Faculty of Medicine, Goethe University Frankfurt, 60590 Frankfurt, Germany; weigert@biochem.uni-frankfurt.de; 3 Frankfurt Cancer Institute (FCI), Goethe University, 60596 Frankfurt am Main, Germany; 4 Translational Research Unit, Member of the German Center for Lung Research (DZL), Thoraxklinik at Heidelberg University, 69126 Heidelberg, Germany; thomas.muley@med.uni-heidelberg.de; 5 Translational Lung Research Center (TLRC) Heidelberg, Member of the German Center for Lung Research (DZL), 69126 Heidelberg, Germany; Mark.kriegsmann@med.uni-heidelberg.de (M.K.); hauke.winter@med.uni-heidelberg.de (H.W.); michael.thomas@med.uni-heidelberg.de (M.T.); 6 Institute of Pathology, University Hospital of Heidelberg, 69126 Heidelberg, Germany; 7 Department of Surgery, Thoraxklinik, University Hospital Heidelberg, 69126 Heidelberg, Germany; 8 Institute for Genetics, Member of the German Center for Lung Research (DZL), Justus Liebig University, 35392 Giessen, Germany; reinhard.dammann@gen.bio.uni-giessen.de; 9 Comprehensive Pneumology Center (CPC) and Institute for Lung Biology and Disease (iLBD), Helmholtz Center Munich-German Research Center for Environmental Health (HMGU), Member of the German Center for Lung Research (DZL), 81377 Munich, Germany; stathopoulos@helmholtz-muenchen.de; 10 Department of Oncology, Thoraxklinik, University Hospital Heidelberg, 69126 Heidelberg, Germany; 11 Department of Internal Medicine, Member of the German Center for Lung Research (DZL), Member of the Cardio-Pulmonary Institute (CPI), Justus Liebig University, 35392 Giessen, Germany; andreas.guenther@innere.med.uni-giessen.de (A.G.); friedrich.grimminger@innere.med.uni-giessen.de (F.G.); 12 Institute for Lung Health (ILH), Justus Liebig University, 35392 Giessen, Germany

**Keywords:** lung cancer, adenocarcinoma, tumor microenvironment (TME), type I interferons (IFNs), interferon regulatory factor 9 (IRF9), versican (VCAN)

## Abstract

**Simple Summary:**

Lung cancer is the leading cause of cancer-related deaths worldwide, accounting for more than 1.6 million deaths per year. The tumor microenvironment (TME) has been shown to play a crucial role in tumor progression and metastasis, and transcription factors link TME signaling to oncogenesis. Type I interferons (IFNs) are strong immune modulators that possess antiproliferative and proapoptotic properties. In this study, we investigated the role of the transcription factor interferon regulatory factor 9 (IRF9) in the IFN pathway in lung cancer. We performed in vitro and in vivo experiments to reveal the oncogenic properties of IRF9, which was highly upregulated in lung adenocarcinoma. For the first time, we showed that IRF9 binds to the promoter of the known oncogene versican, regulates its expression, and thereby promotes oncogenic activity.

**Abstract:**

Transcription factors can serve as links between tumor microenvironment signaling and oncogenesis. Interferon regulatory factor 9 (IRF9) is recruited and expressed upon interferon stimulation and is dependent on cofactors that exert in tumor-suppressing or oncogenic functions via the JAK-STAT pathway. IRF9 is frequently overexpressed in human lung cancer and is associated with decreased patient survival; however, the underlying mechanisms remain to be elucidated. Here, we used stably transduced lung adenocarcinoma cell lines (A549 and A427) to overexpress or knockdown *IRF9*. Overexpression led to increased oncogenic behavior in vitro, including enhanced proliferation and migration, whereas knockdown reduced these effects. These findings were confirmed in vivo using lung tumor xenografts in nude mice, and effects on both tumor growth and tumor mass were observed. Using RNA sequencing, we identified versican (*VCAN*) as a novel downstream target of IRF9. Indeed, *IRF9* and *VCAN* expression levels were found to be correlated. We showed for the first time that IRF9 binds at a newly identified response element in the promoter region of *VCAN* to regulate its transcription. Using an siRNA approach, VCAN was found to enable the oncogenic properties (proliferation and migration) of IRF9 transduced cells, perhaps with *CDKN1A* involvement. The targeted inhibition of IRF9 in lung cancer could therefore be used as a new treatment option without multimodal interference in microenvironment JAK-STAT signaling.

## 1. Introduction

Lung cancer is the leading cause of cancer-related deaths worldwide and accounts for approximately 388,000 deaths annually in Europe [1]. Due to the inconspicuous symptoms associated with late-stage diagnosis, the survival rate of patients with lung cancer is considerably reduced. In the late stages of lung cancer, a combination of available therapeutic options such as surgery, radiation, and chemotherapy can be used as therapy [2]. The tumor microenvironment (TME), which is composed of various cell types and immune cells, is known to play a crucial role in tumor progression and metastasis [3]. Along with numerous cytokines, chemokines, and additional factors secreted by stromal cells, interferons (IFNs), a major family of cytokines, are known to be involved in immune cell activation and modulation [4]. In the TME, IFNs primarily act as tumor-suppressing, antiangiogenic, and proapoptotic cytokines that both directly target tumor cells and enhance the response of other stromal cells [5,6,7,8]. Clinically, IFNs have largely been used as an adjuvant therapy for the treatment of diseases such as malignant melanoma, follicular lymphoma, and chronic myelogenous leukemia. However, severe side effects and the introduction of novel alternative therapies have gradually shifted the role of IFNs to second line or maintenance therapy [9,10].

The interferon regulatory factor (IRF) family comprises nine members (IRF1–9); besides IFN signaling, they play important roles in several processes including inflammation, antiviral response, cell development, and oncogenesis [11,12,13]. For instance, following the stimulation of type I IFNs (e.g., IFNα and IFNβ), transcription factors such as signal transducer and activator of transcription (e.g., STAT1 and STAT2) undergo phosphorylation and, together with IRF9, form a trimeric complex known as interferon-stimulated gene factor 3 (ISGF3). This complex is translocated to the nucleus, binds to the DNA at interferon-stimulated response elements (ISREs), and drives the expression of interferon-stimulated genes (ISGs) [14,15]. IRFs contain a C-terminal IRF-associated domain as well as a well-conserved N-terminal DNA-binding domain, which enables IRF9 to bind to ISREs [16]. Recent studies have shown that IRF9 is associated with unphosphorylated STATs (U-ISGF3) or is expressed independently of STAT1, which leads to altered gene expression profiles [17,18,19,20]. Studies on IRF binding regions have detected dominant clusters for IRF3, IRF5, or IRF9 based on occupancies and revealed that the affinity of IRF9 to ISRE variants is more specific to the sequence 5′-GAAANNGAAACT-3′ [21].

As a major component of the IFN downstream pathway, IRF9 plays a crucial role in viral host defense and inflammation [18,20,22,23,24,25]. Recent studies have indicated that IRF9 is involved in blood vessel formation and angiogenesis [26,27]. However, the role of IRF9 in tumor development and progression remains unclear with the available literature being contradictory. In one study, the combination of IRF9, STAT2, and p65 was shown to induce IL6 expression and inflammation upon IFN stimulation, indicating the tumorigenic activity of IRF9 [28]. However, in prostate cancer, IL6 itself induced IRF9 expression and IRF9 mediated the antiproliferative effects of IFNα2 [29]. This result has been confirmed even in IFNα-resistant cells, such as in human glioblastoma multiforme [30]. In a renal cell adenocarcinoma study, IRF9 knockdown was shown to increase tumor formation in a xenograft model, whereas the overexpression of both IRF9 and STAT2 reduced tumor growth [31]. In lung adenocarcinoma (LUAD), IRF9 induced PD-L1 upregulation upon IFNβ stimulation, which indicates the presence of an immunosurveillance escape mechanism [32]. However, the mechanism by which IRF9 influences tumor development and progression in lung cancer remains unclear. Considering the clinical relevance of IRF9 and the JAK-STAT pathway, which cannot be neglected in the complex TME, this mechanism requires detailed investigation.

The large proteoglycan versican (VCAN), another focus of the present study, is one of the main components of the extracellular matrix and is involved in cell adhesion, proliferation, migration, and angiogenesis [33,34]. In cancer, high expression of the *VCAN* gene is linked with both high malignancy and poor outcomes in patients [34]. Structurally, VCAN contains an N-terminal G1 domain that is able to bind to hyaluronan (HA), a G2 domain with glycosaminoglycan-binding regions (e.g., GAGα and GAGβ), and a G3 domain that contains two EGF-like repeats [34,35]. In breast cancer, VCAN has been shown to regulate proliferation via the G3 domain, whereas the G1 domain is involved in migration processes [36,37,38].

In the present study, we showed that overexpression or reduced expression of *IRF9* increases or decreases, respectively, the cancerous behavior of LUAD cells in vitro and in vivo. We sequenced these characterized cells and showed that *VCAN* and *IRF9* expression are correlated. Indeed, we demonstrated the IRF9-dependent regulation of *VCAN* for the first time and showed that *CDKN1A* might be involved in lung cancer development and progression.

## 2. Results

### 2.1. IRF9 Is Highly Upregulated in Patients with Human LUAD and Is Associated with Decreased Survival

**Figure 1:**
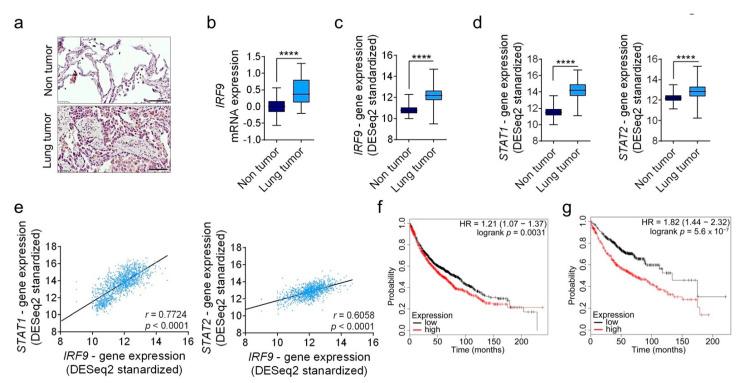
IRF9 expression is upregulated in patients with lung cancer and is associated with decreased survival. (**a**) Representative images of human lung sections stained with IRF9 antibody in LUAD (*n* = 14) versus healthy tissues (*n* = 3). Scale bar: 100 µm. (**b**) mRNA expression of *IRF9* from LUAD and adjacent non-tumor tissues (*n* = 11). (**c**,**d**) Gene expression profiles of (**c**) *IRF9*, as well as (**d**) *STAT1* and *STAT2*, in non-tumor tissues (*n* = 287) and patients with LUAD (*n* = 573) from the TCGA TARGET GTEx study. (**e**) Scatter plots of *IRF9* expression correlated with *STAT1* or *STAT2* expression in 1122 lung tumor samples from a TCGA dataset. The *r* value and two-tailed *p* value were calculated using Pearson’s rank correlation coefficients. (**f**,**g**) Kaplan–Meier curves for *IRF9* expression associated with the survival of patients with (**f**) lung cancer (*n* = 1926) and (**g**) LUAD (*n* = 720) patients, divided by the median into high and low expression. Data are expressed as means ± standard error of the mean. Analysis was performed using Student’s *t*-test: **** *p* < 0.0001, LUAD compared to non-tumor tissues.

We stained a tissue microarray obtained from patients with lung cancer and found that IRF9 was expressed in both tumor and stromal cells in different types of lung cancers, including LUAD (Figure 1a), adenosquamous cell carcinoma, squamous cell carcinoma, small-cell carcinoma, and bronchioloalveolar carcinoma (Figure S1). Furthermore, we found that the mRNA expression of *IRF9* was increased in human non-tumor and lung tumor patient samples (Figure 1b). On analyzing data from The Cancer Genome Atlas (TCGA), we found expression levels of *IRF9* (Figure 1c), *STAT1*, and *STAT2* in patients with lung cancer (Figure 1d), and *IRF9* expression levels were positively correlated with those of *STAT1* and *STAT2* (Figure 1e). Kaplan–Meier curve analysis for lung cancer survival revealed that high *IRF9* expression levels were found to be associated with lower overall survival in patients with lung cancer (HR = 1.21, *p* = 0.0031) and LUAD patients (HR = 1.82, *p* < 0.001) (Figure 1f,g).

### 2.2. IRF9 Is Expressed Upon IFN Treatment and Regulates Tumor Cell Proliferation and Migration

**Figure 2:**
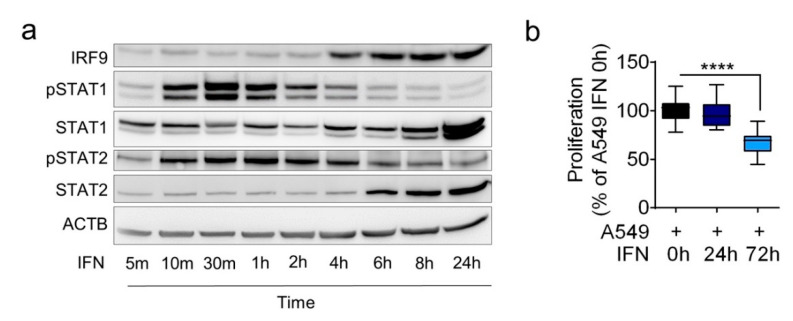
IRF9 is expressed upon IFN stimulation and regulates the proliferative behavior of LUAD cells. Adenocarcinoma A549 cells were treated with 100 U/mL of type I IFN for the indicated time period to evaluate (**a**) intracellular protein levels and (**b**) proliferation as a percentage of the control (*n* = 3). Data are expressed as means ± standard error of the mean. Analysis was performed using one-way analysis of variance with Welch’s *t*-test: **** *p* < 0.0001 (*n* = 3).

The transcription factor complex ISGF-3 was activated following IFN stimulation; hence, we stimulated A549 cells with type I IFN for several periods to visualize the temporal sequence. In Western blot analysis, STAT1 and STAT2 were phosphorylated at an early time point (strongest approximately 30–60 min), whereas the overall increase in the protein levels of IRF9, STAT1, and STAT2 occurred after several hours when phosphorylation was generally diminished (Figure 2a). Notably, the antiproliferative effect of IFN was not observed after 24 h of stimulation but rather after 72 h (Figure 2b). Additionally, the upregulation of *IRF9* mRNA expression with a strong nuclear translocation in A549 cells was observed (Figure S2a–c).

**Figure 3:**
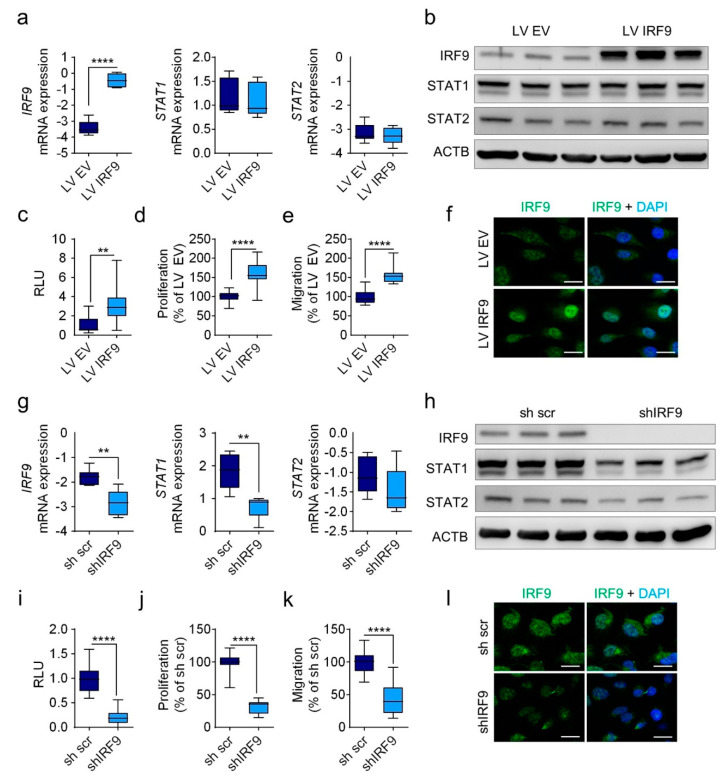
IRF9 overexpression and silencing of regulates the proliferative and migratory behavior in lung adenocarcinoma cells. Using lentiviral particles, A549 cells were transduced to overexpress (A549 LV IRF9) or knock down (A549 shIRF9) *IRF9*. An empty vector (A549 LV EV) or scrambled sequence (A549 sh scr) was used as the respective control. (**a**,**g**) The mRNA expression levels of the indicated genes in the transduced cell lines were evaluated using RT-qPCR and the protein levels (**b**,**h**) by Western blot analysis. (**c**,**i**) IRF9 activity in relative light units (RLU) was measured using a luciferase reporter gene assay with an interferon stimulated response element (ISRE)-containing luciferase vector. (**d**,**j**) Proliferation and (**e**,**k**) migration in the transduced A549 cells were evaluated as a percentage of the control. (**f**,**l**) Immunocytochemistry staining of IRF9 (green) was revealed by an AlexaFlour 488 secondary antibody in transduced cell lines, and counterstained with DAPI (blue). Scale bars: 50 µm. Data are expressed as means ± standard error of the mean. Analysis performed using one-way analysis of variance with Welch’s *t*-test: ** *p* < 0.01; **** *p* < 0.0001 (*n* = 3).

In further analysis, we used a lentiviral approach to stably transduce cells with an overexpression vector (LV IRF9) or a knockdown shRNA vector (shIRF9) together with respective control plasmids (LV EV; sh scr) in the LUAD cell lines A549 and A427. We confirmed the expression of IRF9, STAT1, and STAT2 in A549 cells at the mRNA (Figure 3a,g) and protein (Figure 3b,h) levels. Importantly, *IRF9* knockdown in A549 cells led to decreased expression of STAT1 and STAT2 (Figure 3g), whereas its overexpression did not increase STAT1 and STAT 2 expression (Figure 3a).

To investigate the functional effects of IRF9, we transfected A549 cells with reporter vector pGL4.45, which contains five copies of ISREs followed by the luciferase reporter gene. We found that IRF9 expression was correlated with activity in the luciferase assay indicating a functional effect of IRF9 manipulation (Figure 3c,i). In addition, immunocytochemical staining for IRF9 showed increased IRF9 expression; however, after silencing, IRF9 expression was decreased (Figure 3f,l). In an investigation of tumor behavior, *IRF9* overexpression led to increased proliferation (Figure 3d) and migration (Figure 3e), whereas *IRF9* knockdown resulted in reduced proliferation (Figure 3j) and migration (Figure 3k). Considering the changes in expression, reporter activity, and nuclei enrichment, we found that IRF9 transduction led to functional changes in A549 cells. Additionally, stimulation with IFN revealed that current IRF9 levels affected the upregulation of IRF9 expression (Figure S2e–h). We also confirmed the tumor-promoting and tumor-inhibiting properties (i.e., proliferation and migration) of *IRF9* via its overexpression or silencing, respectively, in A427 cells (Figure S3a–h).

### 2.3. RNA-Seq Revealed VCAN as the Novel Target of IRF9 That Is Correlated with Expression Levels in LUAD Cells

**Figure 4:**
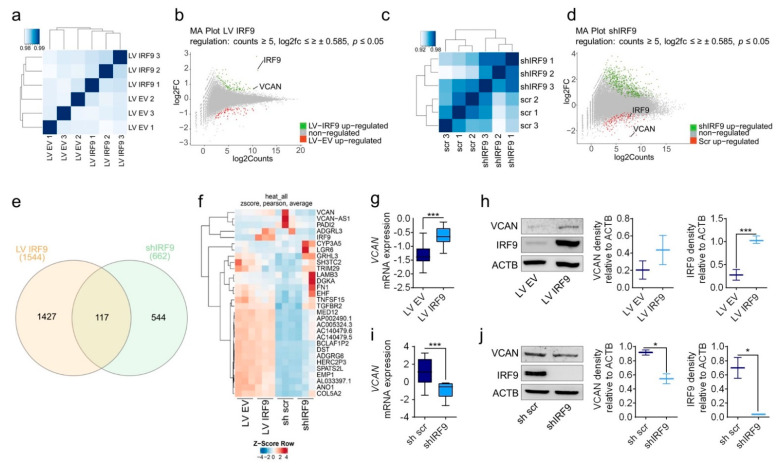
Following manipulation of IRF9, *VCAN* and *IRF9* expression were correlated in LUAD cells. RNA-seq of transduced A549 cells was performed: A549 LV IRF9 cells were compared with A549 LV EV and A549 shIRF9 with A549 sh scr cells, respectively. (**a**,**c**), For correlations of RNA samples, only significantly regulated genes (*p* < 0.05) were selected for further analysis. (**b**,**d**) MA plots of upregulated and downregulated genes from each set (*p* < 0.05), with *IRF9* and *VCAN* highlighted. (**e**) Venn diagram of A549 LV IRF9 cells and shIRF9 regulated genes. (**f**) Heatmap of the top 30 genes significantly regulated in both gene sets in opposite directions. *VCAN* was selected as a potential target of IRF9, and its expression was evaluated at (**g**,**i**) the mRNA and (**h**,**j**) protein expression levels, with quantification in IRF9-overexpressing and -silenced cells (*n* = 6). Data are expressed as means ± standard error of the mean. Analysis was performed using one-way analysis of variance with Welch’s *t*-test: * *p* < 0.05; *** *p* < 0.001.

*IRF9*-overexpressing and -silenced cells were sequenced and samples were correlated to identify the genes involved in the oncogenic property of IRF9 (RNA-seq; Figure 4a,c). Genes that were significantly (*p* < 0.05) regulated were considered for further analysis (Figure 4b,d). In A549 LV IRF9 cells, A549 shIRF9 cells, and both cell sets, 1544, 544, and 117 genes were significantly regulated, respectively (Figure 4e). In addition, we performed gene set enrichment analysis of the KEGG pathway in IRF9-overexpressing and knockdown A459 cells. IRF9 overexpression/knockdown lead to upregulation of pathways e.g., metabolic, lysosome, focal adhesion, ECM receptor interaction and PI3K-Akt signaling (Figure S4). To find a mutual target, we exclusively screened for genes regulated in both sets of transduced A549 cells and particularly those regulated in the opposite directions (Figure 4f). For several regulated genes, i.e., *CDK4*, *CDK6*, *DUSP26*, *EMP1*, *IGFBP5*, *SH3TC2*, and *TRIM29*, we performed RT-qPCR to verify the RNA-seq results and confirm their regulation in transduced A549 cells (Figure S5a,b). Importantly, we identified *VCAN* as a gene of major interest, potentially regulated by IRF9 and responsible for the observed changes in proliferation and migration. We confirmed the expression of VCAN at the mRNA and protein levels in IRF9-overexpressing and -silenced A549 cells (Figure 4g–j) and A427 cells (Figure S6a–d).

### 2.4. VCAN Expression Is Regulated by IRF9 and Is Associated with Lower Overall Survival in Patients with Lung Cancer

**Figure 5:**
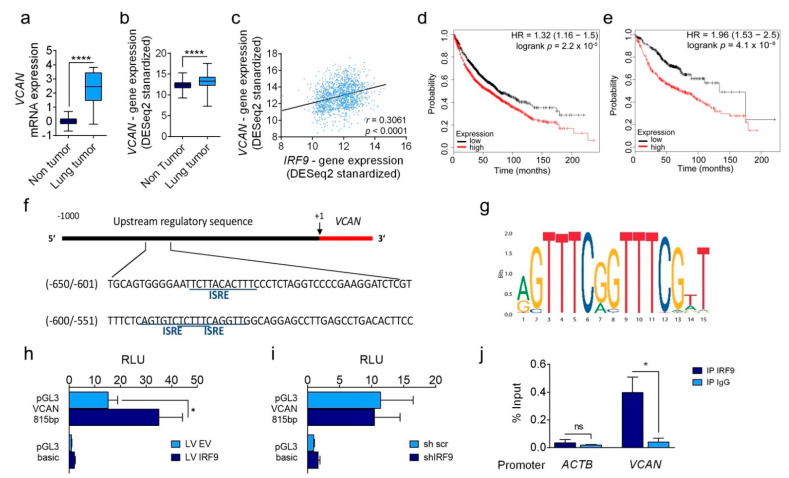
*VCAN* expression is regulated by IRF9 and is associated with lower overall survival in patients with lung cancer. (**a**) mRNA expression of *VCAN* in LUAD and non-tumor tissues (*n* = 11). (**b**) *VCAN* gene expression of lung tissues (*n* = 287) and patients with LUAD (*n* = 573) from the TCGA TARGET GTEx study. (**c**) Scatter plot of *IRF9* expression correlated with *VCAN* expression in lung tumor samples from a TCGA dataset (*n* = 1122). The *R* value and two-tailed *p* value were calculated using Pearson’s rank correlation coefficient. (**d**,**e**) Kaplan–Meier curves of *VCAN* expression in all classes of patients with lung cancer (**d**; *n* = 1926) and LUAD (**e**; *n* = 720). Kaplan–Meier curves show the median divided into high and low expression. (**f**) Sequence of the *VCAN* promoter and potential ISRE based on a FIMO search are underlined. (**g**) IRF9 binding motif from open-access database JASPAR (matrix ID MA0653.1). (**h**,**i**) Transfection of pGL3 VCAN 818 bp showed higher luciferase activity in relative light units RLU 1 = pGL3 control (**h**) A549 LV IRF9 was correlated with A549 LV EV, whereas no difference was noted between (**i**) A549 shIRF9 and A549 sh scr (*n* = 3). (**j**) Chromatin-immunoprecipitation was performed using an antibody against IRF9 and the respective IgG in naïve A549 chromatin. Purified DNA was analyzed using RT- qPCR in the promoter of *ACTB* and *VCAN* (*n* = 3). Data are expressed as means ± standard error of the mean. Analysis was performed using Student’s *t*-test or one-way analysis of variance with Welch’s t-test: * *p* < 0.05; **** *p* < 0.0001.

We investigated VCAN as a potential target of IRF9. First, we detected higher *VCAN* expression in lung cancer samples at the mRNA level (Figure 5a), which was confirmed by TCGA lung cancer database (Figure 5b). Based on the human lung cancer data, a positive correlation was found between *IRF9* and *VCAN* expression (Figure 5c). Moreover, the data revealed that *VCAN* expression was negatively correlated with overall survival in all patients with lung cancer (Figure 5d) and lung adenocarcinoma patients (Figure 5e).

We further investigated the potential regulatory role of IRF9 in *VCAN* expression by using the IRF9 motif and online tool FIMO to detect potential binding sites of IRF9 in the promoter upstream of *VCAN* gene (Figure 5f,g). One potential binding site was located between −636 and −625 bp and, interestingly, two potential ISREs between −593 and −576 bp upstream were overlapping. To confirm binding, we cloned the *VCAN* promoter region from –24 to −842 bp into pGL3 luciferase vector to include the ISRE sites. Following transfection of this vector into our transduced cell lines, A549 LV IRF9 showed higher luciferase transcription activity than A549 LV EV (Figure 5h), whereas transfection into knockdown cells did not alter the previously observed effects (Figure 5i). Furthermore, chromatin-immunoprecipitation in naïve A549 cells revealed that IRF9 binding occurred within the area in which computational binding sites were identified (Figure 5j).

### 2.5. VCAN Knockdown Diminishes the Oncogenic Properties of IRF9 and Increases CDKN1A Expression

**Figure 6:**
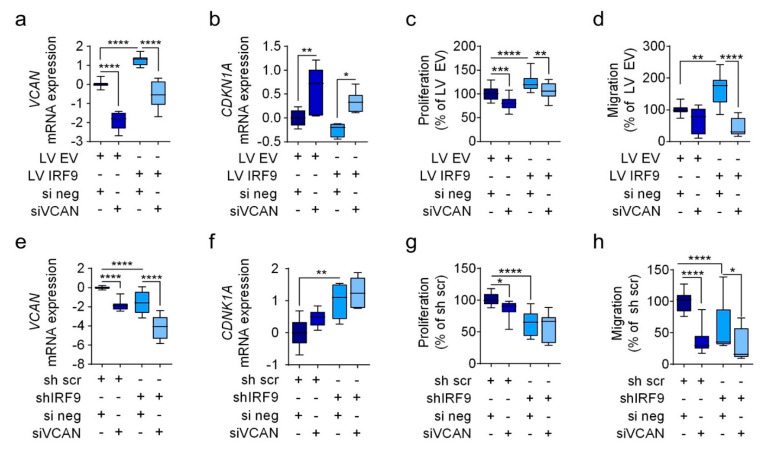
*VCAN* knockdown diminishes the oncogenic features of IRF9. Transduced A549 IRF9 cells were transfected with siRNA against VCAN (siVCAN) to knockdown *VCAN* expression. A non-targeting sequence was used as a transfection control (si neg). (**a**,**e**) mRNA expression of *VCAN* in LV IRF9 and shIRF9 cells; mRNA expression was calculated as ΔΔCt (*n* = 4). (**b**,**f**) mRNA expression of *CDKN1A* in LV IRF9 and shIRF9 cells; mRNA expression was calculated as ΔΔCt (*n* = 4). (**c**,**d**) Proliferation and migration of siVCAN in A549 LV IRF9 cells (*n* = 3). (**g**,**h**) Proliferation and migration of siVCAN in A549 shIRF9 cells (*n* = 3). Data are shown as means ± standard error of the mean. Analysis was via one-way ANOVA: * *p* < 0.05; ** *p* < 0.01; *** *p* < 0.001; **** *p* < 0.0001.

To ascertain whether VCAN regulation is responsible for the oncogenic behavior of IRF9, we used siRNA to knockdown VCAN in the IRF9 transduced cells (i.e., A549 and A427 cells). We successfully confirmed *VCAN* knockdown at the mRNA level in LV IRF9 and shIRF9 (Figure 6a,e). Following VCAN siRNA knockdown, we observed a reduction in the proliferation and migration of A549 LV IRF9 caused by IRF9 overexpression (Figure 6c,d). Moreover, using IRF9-silenced A549 cells, the transfection of siRNA against VCAN further reduced *VCAN* expression (Figure 6e) as well as proliferation and migration abilities (Figure 6g,h). Additionally, there was an increase in *CDKN1A* expression (Figure 6b,f), which suggests that proliferation and migration may be further regulated in a potential *IRF9-VCAN-CDKN1A* axis. Further, we screened mRNA expression of genes involved in the EGF pathway, cell cycle, and apoptosis, such as *TP53*, *CDKN1B*, *CLDN1*, *EGFR*, *CDK4*, *CDK6*, *CCNB1*, *BCL2*, *BAD*, *BIK*, and *CASP3* in A549 cells transfected with siVCAN in the presence of IRF9 overexpression (LV IRF9) or knockdown (sh IRF9). Unfortunately, we could not detect any changes in the expression of *TP53*, *CDKN1B*, *CLDN1*, *EGFR*, *CDK4*, *CDK6*, *CCNB1*, *BCL2*, *BAD*, *BIK*, and *CASP3* (Figure S7a–d).

### 2.6. IRF9 Overexpression and Knockdown of Affected Tumor Growth in the Xenograft Model of Lung Cancer

**Figure 7:**
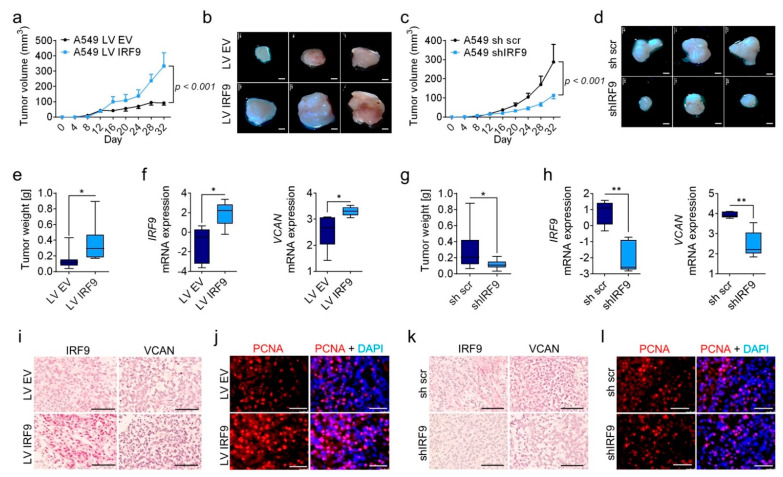
IRF9 overexpression and knockdown influenced tumor growth in a xenograft LUAD tumor model. A549 IRF9 transduced tumor cells were subcutaneously injected into BALB/c nude mice. (**a**) The tumor size of IRF9-overexpressing and empty vector tumors (*n* = 8–10) was measured every four days using calipers. (**b**,**d**) Representative images of dissected tumors. Scale bar: 2 mm. (**e**) The tumor weights of dissected IRF9-overexpressing and empty vector tumors were measured at 32 days. (**f**) *IRF9* and *VCAN* mRNA levels from IRF9-overexpressing and empty vector tumors (*n* = 5). (**i**) IRF9 and VCAN representative immunohistochemistry images from IRF9-overexpressing and empty vector tumors. Scale bar: 100 µm. (**j**) Immunofluorescence staining of PCNA from *IRF9*-overexpressing and empty vector tumors. Scale bar: 50 µm. (**c**) Tumor volume of IRF9-silenced and control tumors were measured every four days using calipers (*n* = 8–10). (**d**) Representative images of dissected tumors. Scale bar: 2 mm. (**g**) Tumor weight of dissected IRF9-silenced and control tumors measured at 32 days. (**h**) *IRF9* and *VCAN* mRNA levels from IRF9-silenced and control tumors (*n* = 5). (**k**) IRF9 and VCAN representative immunohistochemistry images from IRF9-silenced and control tumors. Scale bar: 100 µm. (**l**) Immunofluorescence staining of PCNA from IRF9-silenced and control tumors. Scale bar: 50 µm. Data are expressed as means ± standard error of the mean. Analysis was performed using Student’s *t*-test: * *p* < 0.05, ** *p* < 0.01.

To investigate the role of IRF9 in vivo, we injected the human transduced A549 cell lines into immunodeficient BALB/c nude mice. In accordance with in vitro results, IRF9-overexpressing cells showed increased tumor growth (Figure 7a,b) and tumor weight (Figure 7e). To confirm the stable overexpression of *IRF9* and the associated target gene *VCAN*, we isolated RNA from tumor homogenate. We found that *IRF9* and *VCAN* expression in IRF9-overexpressing tumors (Figure 7f) was increased relative to that in control tumors. Furthermore, at the mRNA level in IRF9-overexpressing tumors, we found increased levels of *STAT1*, *STAT2*, and *CDKN1A*, as well as an increase in the level of the proliferation marker *PCNA* (Figure S8a). In accordance with tumor staining results, we observed increased levels of IRF9 and VCAN in IRF9-overexpressing tumors (Figure 7i); PCNA staining also showed an increase in PCNA expression in these tumors (Figure 7j). In addition, injection of shIRF9 cells showed reduced tumor growth (Figure 7c,d)and tumor weight (Figure 7g) in the IRF9-knockdown group. The downregulation of *IRF9* and *VCAN* expression was conserved until tumor excision (Figure 7h), *STAT1*, and *CDKN1A* expression showed decreased tendency and *STAT2*, and *PCNA* expression were significantly reduced in the *IRF9*-knockdown group (Figure S8b). Finally, shIRF9 tumors showed decreased expression of IRF9, VCAN, and PCNA compared with that in the control (Figure 7k,l).

## 3. Discussion

In this study, we showed for the first time the oncogenic effects of IRF9 in lung cancer. On combining the in vitro and in vivo results, we revealed that: (i) IRF9 expression was significantly increased in human lung cancer samples; (ii) IRF9 overexpression increased tumor cell proliferation and migration in vitro; (iii) IRF9 silencing decreased tumor cell proliferation and migration in vitro; (iv) *VCAN* is a direct downstream target of IRF9; (v) IRF9 overexpression increased lung tumor growth in vivo; and (vi) IRF9 silencing reduced lung tumor growth in vivo.

In both patients with lung cancer and LUAD patients, Kaplan–Meier curves showed that reduced survival was associated with IRF9 overexpression. These findings are confirmed by corroborating human survival data, in which low IRF9 expression was shown to be beneficial patients with lung cancer [28]. This holds also true for ovarian cancer, gastric cancer and ER positive breast cancer (Figure S1). However, IRF9 has been shown to confer chemoresistance in breast cancer, and suggested as a prognostic marker for chemotherapy and overall survival in triple-negative breast cancer (TNBC), in which high IRF9 levels are associated with a better outcome [39,40]. Studies showed that the loss of IRF9 in TNBC accompanied a reduced response to intratumoral IFN signaling, whereas the presence of IRF9 could enhance antitumoral immunity and enhance patient survival [40]. However, in vitro breast cancer data might differ. In the breast cancer cell line MCF7, which does not originate from TNBC and was still able to respond to IFN treatment with reduced proliferation, the overexpression of IRF9, but not STAT1 or STAT2, increased the resistance of cells against the chemotherapeutic drug paclitaxel [39]. Based on these findings, it should not be generalized that IRF9 is beneficial in all patients with breast cancer.

In a study of patients with clear cell RCC, patients with nuclei positive for IRF9 had a better prognosis than those with absent IRF9 expression. Similarly, to our findings in patients with lung cancer, the status of STAT2 did not influence survival in these patients [31]. In acute myeloid leukemia and prostate cancer, IRF9 acts as a tumor suppressor; in pancreatic diseases, it acts as an oncogene [29,41,42]. In the present study, in which we focused on lung cancer cells, IRF9 clearly promoted cell proliferation, migration, and tumor growth. It is clear that, in patients with lung cancer, the tumor stromal cells secrete IFNs to activate immune defense and inhibit tumor growth, as previously described [5]. These results support findings that long-term stimulation with IFNs reduces canonical signaling [18]. With the loss of IFN sensitivity, IRF9 is no longer able to influence the antiproliferative effects of IFN; consequently, the downstream profile of IRF9 changes. In these circumstances, it is reasonable that Kaplan–Meier curves show lower overall survival of patients with lung cancer when IRF9 expression is high.

In tumor cells, the role of IRF9 appears complex. Some studies reported the tumor suppressive properties of IRF9, such as the facilitation of the antiproliferative effects of IFN in prostate cancer cells, reduced tumor growth in renal clear cell carcinoma, and effects in leukemia [29,31,41]. However, other studies highlighted the oncogenic properties of IRF9. Together with STAT2 and p65, IRF9 enhances lung cancer cell growth and, in pancreatitis, promotes proliferation and migration [28,42]. Persistent stimulation with IFNs, for example, is known to change the signaling cascade toward noncanonical signaling with reduced phosphorylation status (U-ISGF3) and high IRF9 levels [17,18]. The effects of U-ISGF3 on DNA damage resistance and the fact that IRF9 overexpression leads to resistance against several chemotherapeutics indicates that IRF9 could affect tumor therapy, which in turn helps explain the oncogenic properties of IRF9 [18,39,43]. Vascular smooth muscle cells (VSMCs) are another cell type of interest. VSMCs are activated by IRF9 to facilitate vessel repair and neovascularization, thereby supporting tumor development with angiogenesis [26,27]. It is possible that the loss of IRF9 reduces neoangiogenesis and further the supply of oxygen and nutrients. Using conditional or global IRF9-knockout animals will enable the relevant cell types to be harvested and important experiments to be conducted in this field.

Our work is the first to both describe ISREs and show the direct binding of IRF9 to the promoter of *VCAN*. Because we focused mainly on the functional effects of IRF9 and VCAN, it would be of interest to investigate their regulation in greater detail in future research. Although U-ISGF3 is postulated to be responsible for the observed VCAN regulation, the principle binding of IRF9 in the absence of phosphorylation does not verify this theory beyond all doubt. Overall co-immunoprecipitations with IRF9 in cells with manipulated expression could reveal how IRF9 is recruited in this context, and additional ChIP experiments against STAT1 and STAT2 are necessary to clarify in which complex IRF9 binds to the DNA and if other factors are responsible, as previously described for p65 [28].

The roles of VCAN in cancer are diverse, ranging from activation of the TME to tumor cell effects [44]. In our siRNA experiments, *VCAN* knockdown led to an increase in *CDKN1A* expression, which was correlated negatively with *VCAN* mRNA levels. The interplay of VCAN with its EGF-like domain, p53, and *CDKN1A* has been shown to be dependent on oncogenic mutations in *EGFR*. When *EGFR* is mutated, the ligand EGF facilitates an unexpected upregulation of the originally tumor-suppressing gene *CDKN1A*, which enables further cell proliferation and tumor progression [45,46]. This has been confirmed particularly in patients with lung cancer, in which high expression levels of *CDKN1A* with wild-type *EGFR* status was associated with better survival, whereas *CDKN1A* expression with oncogenic *EGFR* mutations was correlated with poor outcome [47]. With the p53 null mutation, *CDKN1A* activity does not lead to senescence but rather promotes cancer behavior [48,49]. In our study, A549 cells carried both EGFR and p53 wild-types; thus, *CDKN1A* could serve as a cyclin-dependent kinase inhibitor and the siVCAN-dependent upregulation of *CDKN1A* might explain the observed reduction in cell proliferation and migration.

In human breast tumors, VCAN is enriched in proliferating tumor areas and particularly in HA-rich portions. When HA-binding affinity at the G1 domain of VCAN is lacking, *CDKN1A* expression in embryonic fibroblasts is upregulated [50,51]. Although IRF9 is not reported to influence cell cycle progression in cancer cells, the observed changes in *CDKN1A* expression alterations encourage research focused in this field. In addition, investigating the role of IRF9 in a VCAN-dependent manner in oncogenic p53 or EGFR cells would be of further interest.

Overall, our findings show that IRF9 directly binds to the promoter of *VCAN* and activates its expression. Subsequently, the G3 domain of *VCAN* with its EGF-like motif becomes a key player in the regulation of proliferation and migration via the downstream regulation of *CDKN1A*.

## 4. Materials and Methods

### 4.1. Acquisition of Human Tumor Data

We analyzed 1926 lung tumors, including 866 LUADs that had been profiled by Affymetrix microarray analysis (www.kmplot.com) for Kaplan–Meier analysis [52]. Accordingly, *IRF9* (probe set ID: 203882-at) and *VCAN* (probe set ID:204620_s_at) expression levels were divided at the median into high and low-expression subgroups. Overall survival analyses for all lung tumors or LUAD by Kaplan–Meier and Cox proportional hazard analyses were performed. The dataset used for the comparison of *IRF9*, *STAT1*, *STAT2*, and *VCAN* expression in lung tumor vs. non-tumor tissues was obtained from the UCSC Xena on 11/29/19 as DESeq2 standardized and includes GTEx and TCGA data [53]. RNA samples from human LUAD tissues were obtained from the Lung Biobank Heidelberg, a member of the BioMaterialBank Heidelberg and the Biobank platform of the German Center for Lung Research (DZL). The lung cancer tissue array LUC1501 contains 150 cores from normal/benign (three cases) and cancers (70 cases with grading and TNM staging data), and duplicated cores per case were purchased from Pantomics, Inc. (cat no. LUC 1501; Richmond, CA, USA). The tumor specimens were presented in duplicate for internal control and to assess tumor heterogeneity. In addition, a pathologist validated the tumors in the cores. All specimens were analyzed using a slide scanner NDP Nanozoomer 2.0HT (Hamamatsu-Photomics, Hamamatsu, Japan) and its viewing platform (NDP.Viewer).

### 4.2. Protein Extraction and Western Blot Analysis

Cells were washed with ice-cold PBS at pH 7.4 (Gibco, 10010056, Life Technologies, Carlsbad, CA, USA) before being harvested by scraping directly with RIPA lysis buffer (SCBT, sc-24948, Santa Cruz Biotechnology, Dallas, TX, USA) supplemented with Complete Protease Inhibitor Cocktail (Roche, 11697498001, Roche, Basel, Switzerland), PMSF (Sigma-Aldrich, 93482-250ML-F, St. Louis, MO, USA), and sodium orthovanadate (NEB, P0758S, New England Biolabs, Frankfurt, Germany), and tumor tissue was disrupted with ceramic beads. Cell lysates were centrifuged to remove cell debris. Protein concentration was measured before dilution and denaturation by heat and treatment with 2-mercaptoethanol (Carl Roth, 4227.1, Carl Roth, Karlsruhe, Germany). Cell lysates were separated on hand-made polyacrylamide gels and blotted on nitrocellulose membranes or Immun-Blot PVDF Membranes (Bio-Rad, 1620112; 1620177, Bio-Rad, Hercules, CA, USA). After incubation with primary antibodies at 4 °C overnight and following an additional 1 h of incubation with anti-Rabbit or anti-Mouse IgG (H + L) HRP-Conjugate (Promega, W4011; W4021, Promega, Madison, WI, USA), proteins were detected using WesternBright ECL (Biozym, 541005X, Biozym, Hessisch Oldendorf, Germany) and an ImageQuant device (GE Healthcare, Chicago, IL, USA). Antibodies were purchased and then diluted in 5% skim-milk (Carl Roth, T145.2) or 5% bovine serum albumin (BSA; Sigma-Aldrich, A2153) in tris-buffered saline with Tween (TBST) as indicated: IRF9, 1:500 (SCBT, sc-10793, sc-365893); STAT1, 1:1000 (BD, 610185, BD, Franklin Lakes, NJ, USA); Phospho-STAT1 (Tyr701), 1:1000 (CST, 9167); STAT2, 1:1000 (SCBT, sc-1668); Phospho-STAT2 (Tyr689), 1:1500 (Merck Millipore, 07-224, Merck KGaA, Darmstadt, Germany); PCNA, 1:2000 (SCBT, sc-7987); VCAN, 1:2000 (Abcam, ab19345, Abcam, Cambridge, UK); ACTB, 1:3000 (Abcam, ab6276); and GAPDH, 1:3000 (Abcam, ab8245).

### 4.3. Immunohistochemistry

The lung tumor tissue array (TMA) described in Section 4.1 was used for immunohistochemical analysis. In preparation for immunohistochemical staining, 3-µm TMA and 3-µm xenograft tumor sections were rehydrated, and antigen-retrieval was achieved with citrate buffer following a previously described procedure [54,55]. The sections were then blocked and incubated with IRF9 1:100 (SCBT, sc-10793) and VCAN 1:150 (Abcam, ab19345) primary antibodies overnight. For detection, IRF9 and VACN antibody-stained sections were washed in 1x PBS, and antibody binding was determined using an ImmPRESS reagent kit (Vector Laboratories, Burlingame, CA, USA) and ZytoChem Plus AP Polymer Kit (Zytomed Systems, Berlin, Germany) according to the manufacturer’s instructions. Sections were embedded using Pertex (Medite Service AG, Dietikon, Switzerland) and scanned using a slide scanner (NDP Nanozoomer 2.0HT).

### 4.4. Immunocytochemical Staining

For immunocytochemical staining, cells were seeded in eight-well glass chamber slides (Sarstedt, Nürnbrecht, Germany) and grown to 70% confluence. Cells were then washed with PBS at pH 7.4, fixed with 4% paraformaldehyde (Sigma-Aldrich, 158127), and treated with 0.3% Triton X-100 (Carl Roth, 3051.3). After blocking with 1% BSA for 1 h, cells were incubated with IRF9 antibody (1:100; SCBT, sc-10793) was applied for 90 min, washed thoroughly, and incubated with goat anti-rabbit IgG Alexa Fluor 488 secondary antibody (1:1000; Thermo Fisher, A27034, Thermo Fisher Scientific, Waltham, MA, USA) was applied for 1 h. The slides were visualized under a confocal microscope (Zeiss LSM 710, Carl Zeiss, Oberkochen, Germany) using Zen 2011 software.

### 4.5. Cell Culture

A549, A427, and HEK293T cells were purchased from American Type Culture Collection (ATCC, Manassas, VA, USA) and maintained in DMEM and RPMI media, respectively supplemented with 10% fetal calf serum (Gibco, 16140071) and 1 U/mL penicillin–streptomycin (Gibco, 15140122). All cell lines were tested for mycoplasma tested using LookOut^®^ Mycoplasma PCR Detection Kit (Merck, MP0035-1KT). For IFN stimulation, cells were treated with 100 U/mL of type-I-Interferon (PBL, 11200-2, PBL Assay Science, Piscataway, NJ, USA). Treatment with PBS at pH 7.4 represented the control.

### 4.6. RNA Isolation, cDNA Synthesis, and RT-qPCR

Cells were washed with PBS at pH 7.4 prior to being harvested by scraping directly with TriZOL reagent (Ambion, 15596018, Thermo Fisher Scientific). RNA isolation was performed according to the manufacturer’s protocol. Subsequently, 1000 ng of total RNA was transcribed into cDNA using a High-Capacity cDNA Reverse Transcription Kit (Thermo Fisher Scientific, 4368813) according to the manufacturer’s instructions. qRT-PCR was performed for diluted cDNA and analysis was performed using a StepOnePlus Real-Time PCR System (Thermo Fisher Scientific) with PowerUp SYBR Green Mastermix (Thermo Fisher Scientific, A25778) at the following conditions: 2 min at 50 °C, 2 min at 95 °C, followed by 40 cycles of 15 s at 95 °C and 1 min at 60 °C. All primer pairs were subjected to a BLAST search with the NCBI primer BLAST tool (https://www.ncbi.nlm.nih.gov/tools/primer-blast/) and purchased from Sigma-Aldrich. To confirm primer specificity, a melting curve was constructed after RT-qPCR was performed and the PCR products were separated on an agarose gel. *IRF9* FP: 5′-CCA TCA AAG CGA CAG CAC AG-3′; *IRF9* RP: 5′-GAG CAC AGA GGG ACT GAG TG-3′; *STAT1* FP: 5′-ATC AGG CTC AGT CGG GGA ATA-3′; *STAT1* RP: 5′-TGG TCT CGT GTT CTC TGT TCT-3′; *STAT2* FP: 5′-CTG CTA GGC CGA TTA ACT ACC C-3′; *STAT2* RP: 5′-TCT GAT GCA GGC TTT TTG CTG-3′; *VCAN* FP: 5′-GAA TGT CAC TCT AAT CCC TGT C-3′; *VCAN* RP: 5′-TGT CTC GGT ATC TTG CTC AC-3′; *CDKN1A* FP: 5′-AGT CAG TTC CTT GTG GAG CC-3′; *CDKN1A* RP: 5′-GCA TGG GTT CTG ACG GAC AT-3′; *HPRT* FP: 5′-TGA CAC TGG CAA AAC AAT-3′; *HPRT* RP: 5′-GGT CCT TTT CAC CAG CAA-3′; *IGFBP5* FP: 5′-TGA CCG CAA AGG ATT CTA CAA G-3′; *IGFBP5* RP: 5′-CGT CAA CGT ACT CCA TGC CT-3′; *CDK4* FP: 5′-CCT CTC TAG CTT GCG GCC TG-3′; *CDK4* RP: 5′-CTC AGA TCA AGG GAG ACC CTC AC-3′; *CDK6* FP: 5′-CAG GGA AAG AAA AGT GCA ATG A-3′; *CDK6* RP: 5′-CGA AGC GAA GTC CTC AAC AC-3′; *GRHL3* FP: 5′-CAG GAG TCG ATG CTC TTC CC-3′; *GRHL3* RP: 5′-CCC AGG GTG TAT TCA AAG TCA C-3′; *DUSP26* FP: 5′-TAA CTG GCT TTG GGC TTC TAT G-3′; *DUSP26* RP: 5′-GAT GTT GAA CGG TTG GCA TCT-3′; *EMP1* FP: 5′-TCT GAT TCC CTT CAT TGT GTG A-3′; *EMP1* RP: 5′-TCC AAA TCA AAC TGA TAG GCA GC-3′; *TRIM29* FP: 5′-CAA GCA CCC TGC GAT GGA-3′; *TRIM29* RP: 5′-GTT GGT GGT CTT GGC ATC CTT-3′; *TP53* FP: 5′-CAG CAC ATG ACG GAG GTT GT-3′; *TP53* RP: 5′-TCA TCC AAA TAC TCC ACA CGC-3′; *CDKN1B* FP: 5′-ATC ACA AAC CCC TAG AGG GCA-3′; *CDKN1B* RP: 5′-GGG TCT GTA GTA GAA CTC GGG-3′; *CLDN1* FP: 5′-TGG AAG ACG ATG AGG TGC AGA AGA-3′; *CLDN1* RP: 5′-CAA CTA AAA TAG CCA GAC CTG CA-3′; *EGFR* FP: 5′-GCG TTC GGC ACG GTG TAT AA-3′; *EGFR* RP: 5′-GGC TTT CGG AGA TGT TGC TTC-3′.

### 4.7. Lentiviral Transduction

HEK293T cells were co-transfected with the lentiviral overexpressing or silencing plasmid, the envelope plasmid pMD2.G, and the packaging plasmid pCMVΔR8.2 (both Addgene, Watertown, MA, USA) using Fugene HD (Promega, E2311) and reduced medium. After 24 h, the medium was replaced with complete medium and viral particles were harvested. After an additional 24 h, they were used to transfect A549 cells using a final concentration of 0.8 mg/mL Polybrene (Merck, TR-1003-G) twice after 6 h. Puromycin (Gibco, A1113803) was used to select and culture transduced A549 cells. Lentiviral constructs for IRF9 overexpression (pLV IRF9) and knockdown (pLKO.1 shIRF9) as well as respective vector controls (pLV EV; pLKO.1 sh scr), as previously described [18], were generously gifted from George R. Stark’s lab (Cleveland Clinic Lerner Research Institute, OH, USA).

### 4.8. Proliferation and Migration Assay

The proliferation assay was performed with previously serum-starved cells using a BrdU colorimetric cell proliferation ELISA (Roche, 11647229001), according to the manufacturer’s protocol, and measured using a microplate reader (Infinite M200 PRO; Tecan, Männedorf, Switzerland). For the migration assay, a similar number of cells was seeded into a Transwell membrane insert with reduced medium (8-µm pore size; Falcon, 353097, BD) and allowed to migrate for 6 h toward the lower compartment containing the complete medium. Thereafter, migrated cells were fixed with methanol, stained with 10% crystal violet (Sigma-Aldrich, V5265), excised, and then fixed onto slides using Pertex mounting medium (Medite Service AG). Slides were scanned using a slide scanner (NDP Nanozoomer 2.0HT). and quantified with Fiji software and ITCN macro (NIH, Bethesda, MD, USA).

### 4.9. RNA-Seq and Bioinformatics Analysis

For RNA-seq, RNA was isolated from three independent viral transduced A549 cells of each construct (A549 LV EV, A549 LV IRF9, A549 sh scr, and A549 shIRF9) using the miRNeasy Micro Kit (Qiagen, Hilden, Germany) combined with on-column DNase digestion (DNase-Free DNase Set, Qiagen) to avoid contamination by genomic DNA. RNA and library preparation integrity were verified with LabChip Gx Touch 24 (Perkin Elmer, Waltham, MA, USA). For Truseq Stranded mRNA Library preparation, 3 µg of total RNA was used as an input following the low sample protocol (Illumina, Berlin, Germany). Sequencing was performed on the NextSeq500 instrument (Illumina) using v2 chemistry; this resulted in a minimum of 25 million reads per library with a 1 × 75 bp single-end setup. The resulting raw reads were assessed for quality, adapter content, and duplication rates using FastQC [56]. Trimmomatic version 0.36 was employed to trim reads after a quality drop below a mean of Q20 in a window of 10 nucleotides [57]. Only reads between 30 and 150 nucleotides were used for further analyses. Trimmed and filtered reads were aligned against the Ensembl human genome version hg38 (GRCh38) using STAR 2.6.0c; mapped length was set to 10% and the parameter “—outFilterMultimapNmax to 999” was used to allow mapping to multiple positions [58]. The number of reads aligning to genes was counted using the featureCounts 1.6.0 tool from the Subread package [59]. Only reads mapping at least partially inside exons were admitted and aggregated per gene. Reads overlapping multiple genes or aligning to multiple regions were excluded. Differentially expressed genes were identified using DESeq2 version 1.18.1 [60]. Only genes for which *p* < 0.05 and a minimum combined mean of five reads existed were considered significantly differentially expressed. The Ensemble annotation was enriched with UniProt data (release 06.06.2014) based on Ensembl gene identifiers [61].

### 4.10. In Silico Promoter Analysis

We used the online tool HOMER v4.10 (homer.ucsd.edu/homer/) to extract the ISRE binding motif [62]. The VCAN promoter was extracted from the online tool Eukaryotic Promoter Database (epd.epfl.ch) from −1000 bp to +100 bp [63]. We used FIMO (Find Individual Motif Occurrences) from the online tool MEME Suite 5.1.1 (meme-suite.org/tools/fimo) to identify binding sites of the IRF9 motif in the *VCAN* promoter; results for which *p* < 0.001 were considered for further analysis [64].

### 4.11. Plasmid Construction and Luciferase Reporter Assay

A 818-bp segment of the upstream promoter sequence of human *VCAN*, including the potential ISRE, was amplified from human A549 DNA using Phusion High-Fidelity DNA Polymerase (NEB, M0530L) and specific primers with an overhanging sequence for restriction enzymes: FP: 5′-ATA TTA CTC GAG GAC TGA AGG AAA GGA AGA ACG AAG-3′ (XhoI); RP: 5′-ATT TAA GCT TTC AGA GCC GAG GAG GAG ACT CA-3′ (HindIII). PCR products were separated on agarose gels and excised bands were purified using GenElute (Sigma-Aldrich, NA1020). The PCR construct and pGL3 basic (Promega) vector were restricted with the aforementioned enzymes, the paternal plasmid was digested with DpnI, ligation was conducted with T4 DNA ligase at a ratio of 1:3, before transformation into 10-beta competent *Escherichia coli* (all NEB: R0176; M0202S; C3019H), and finally incubation overnight on agar plates containing ampicillin (Roche, A0166) at 37 °C. Clones were screened with PCR using the recommended primer pair: GLprimer2: 5′-CTT TAT GTT TTT GGC GTC TTC CA-3′; RVprimer3: 5′-CTA GCA AAA TAG GCT GTC CC-3′. Plasmids of positive clones were purified using peqGOLD Plasmid Miniprep (VWR, #13-6943-02, VWR, Radnor, PA, USA) and confirmed by test restriction and sequencing via Eurofins (Cologne, Germany). pGL4.45 was purchased from Promega; it sequentially contains five ISREs prior to a minimal promoter and the luc2P gene. For the luciferase assay, we used a Dual-Glo Luciferase Assay System (Promega, E2920). Therefore, cells were cotransfected with the firefly luciferase plasmids pGL3 basic, pGL3 VCAN 818 bp, or pGL4.45. The *Renilla* luciferase plasmid pCMV-RL (Promega) was used as the internal control. Cells were lysed with passive lysis buffer and transferred to white 96-well plates. LAR II was applied and firefly luciferase measured using an Infinite M200 PRO microplate reader (Tecan). STOP and Glo was applied and *Renilla* luciferase measured to normalize the activity of firefly luciferase.

### 4.12. Chromatin Immunoprecipitation

A549 cells were crosslinked using 1% formaldehyde (Sigma-Aldrich, F8775) for 10 min and then quenched using a final concentration of 125 mM glycin (Carl Roth, 0079.1) for 5 min. The following steps were conducted on ice using prechilled buffers and solutions, which were supplemented with cOmplete Protease Inhibitor Cocktail (Roche, 11697498001), PMSF (Sigma-Aldrich, 93482-250ML-F), and sodium orthovanadate (NEB, P0758S). After thorough washing with PBS at pH 7.4 (Gibco, 10010056), nuclei were isolated and sonicated by three cycles of 5 s with a tip sonicator (Bandelin Sonopuls, Bandelin, Berlin, Germany) followed by 20 cycles of 30 s on/30 s off with Bioruptor Pico (Diagenode, Seraing, Belgium). After centrifugation, fragment size of 200–500 bp were confirmed using agarose gel electrophoresis, and soluble chromatin was immunoprecipitated using specific antibodies against histone H3 (Abcam, ab12079), IRF9 (SCBT, sc-365893 X), and IgG1 as a control (CST, 5415, Cell Signaling Technology, Danvers, MA, USA). Chromatin–antibody complexes were purified using Protein G Agarose beads (Sigma-Aldrich, 16-201) and a high/low salt buffer (20 mM Tris-HCl, pH 8.0; 2 mM EDTA; 1% NP-40; 0.1% SDS; 0.5 mM/0.15 M NaCl). Chromatin was eluted with 0.1 M NaHCO3, and 1% SDS, followed by RNAse (ThermoFisher, EN0531) and Proteinase K (Sigma-Aldrich, P2308) treatment. DNA was purified using a QIAquick PCR Purification Kit (Qiagen) and subjected to qPCR, in which it was calculated as a percentage of input 100 × 2^(−dCT)^ [dCT = CT ChIP−(CT input−log_2_ dilution factor)]. *ACTB* FP: 5′-AAC CGG ACC GCC GTG-3′; *ACTB* RP: 5′-TCG CGC CTC CGA ACT G-3′; *VCAN* FP: 5′-CTC TTG CTC TAT TTA TGA TCA GCT G-3′; *VCAN* RP: 5′-CTA GTG GAT AGG AGC TGG CAC-3′.

### 4.13. SiRNA Transfection

FlexiTube siRNA targeting *VCAN* (Qiagen ID: SI04948587; target sequence CAT GCG CTA CAT AAA GTC AAA) and AllStars Negative Control siRNA were purchased from Qiagen. Briefly, cells were cultured the day before transfection to 60–70% confluence. The siRNA and control were diluted using OptiMEM (Gibco, 11058021) prior to adding the transfection reagent Hiperfect (Qiagen, ID: 301707). After incubation for 5 min, the transfection mixture was added to the wells containing complete medium. A final siRNA concentration of 2.5 × 10^−9^ mol∙L^−1^ and a final ratio of 1.2 µL Hiperfect per 10^−9^ mol of siRNA were used. For RNA isolation, cells were harvested two days after transfection. For Transwell membrane migration assays, cells were starved for one day after transfection and, after an additional day, seeded for migration. For the cell proliferation assay, starvation medium was used for transfection and then replaced with complete medium after one day. Data were collected after one additional day.

### 4.14. Animal Studies

All animal studies were approved by the relevant authority (Regierungspräsidium Darmstadt, Hessen, Germany; approval no. B2/1062) and performed in accordance with German animal protection laws (TierSchG). Nude mice were purchased from JAX (Jackson Laboratory, Bar Harbor, ME, USA and were housed under specific pathogen-free conditions in individual ventilated cages. For the subcutaneous tumor model, 3 × 10^6^ transduced A549 IRF9 cells were injected subcutaneously in a final volume of 100 µL of 0.9% NaCl (B. Braun, Melsungen, Germany). Tumor growth was monitored as previously described [65]. Mice were sacrificed 30 days (A549) after injection.

### 4.15. Statistical Analysis

Statistical analysis was performed using GraphPad Prism 8 software (GraphPad Software, San Diego, CA, USA). One-way analysis of variance was used to compare the means of individual groups with the control group. To compare two independent groups, unpaired Student’s *t*-tests were used when the same standard deviation was assumed. If the standard deviations were not the same, Welch’s *t*–test was used instead. Data are expressed as means ± standard error of the mean. *p* < 0.05 was considered statistically significant.

## 5. Conclusions

We identified IRF9 as an oncogenic transcription factor in LUAD that facilitated increased proliferative and migratory behaviors. For the first time, we showed the direct binding of IRF9 to the promoter of *VCAN* as well as a correlation between *IRF9* and *VCAN* expression in lung cancer. In addition, siRNA experiments showed that VCAN diminished the tumor promoting effects of IRF9 and indicated the involvement of the tumor suppressor *CDKN1A*.

## Data Availability

Data used in this study for overall survival analysis were found in a publicly accessible repository www.kmplot.com (*IRF9* probe set ID: 203882-at and *VCAN* probe set ID: 204620_s_at). *IRF9* and *VCAN* expression levels were analyzed using UCSC Xena Browser (https://xenabrowser.net/) including GTEx TCGA dataset.

## Supplementary Materials

The following are available online at https://www.mdpi.com/2072-6694/13/2/208/s1, Figure S1: IRF9 expression in different types of lung cancer types and its association with survival in various cancer types, Figure S2: IFN stimulation leads to increased expression and activity of IRF9; Figure S3: IRF9-transduced A427 cells have an oncogenic phenotype, Figure S4: Gene set enrichment analysis of IRF9 overexpressing and silenced A549 cells, Figure S5: Validation of RNA-seq target genes in transduced A549 cells, Figure S6: VCAN expression in IRF9-transduced A427 lung cancer cells, Figure S7: Knockdown of VCAN does not alter genes that are involved in EGF, cell cycle and apoptosis pathways, Figure S8: Evaluation of IRF9-associated genes in IRF9-overexpressing or -silenced tumors.
