# Supplementary material for: Interferon Regulatory Factor 9 Promotes Lung Cancer Progression via Regulation of Versican
Source: Cancers (Basel). 2021 Jan 8;13(2):208. doi: 10.3390/cancers13020208 (PMC7827113; doi:10.3390/cancers13020208)
Supplement: Supplementary file 1 [file cancers-13-00208-s001.pdf]

Supplementary material

Interferon Regulatory Factor 9 Promotes Lung Cancer Progression via Regulation of Versican

David Brunn, Kati Turkowski, Stefan Günther, Andreas Weigert, Thomas Muley, Mark Kriegsmann, Hauke Winter, Reinhard H. Dammann, Georgios T Stathopoulos, Michael Thomas, Andreas Guenther, Friedrich Grimminger, Soni S. Pullamsetti, Werner Seeger and Rajkumar Savai

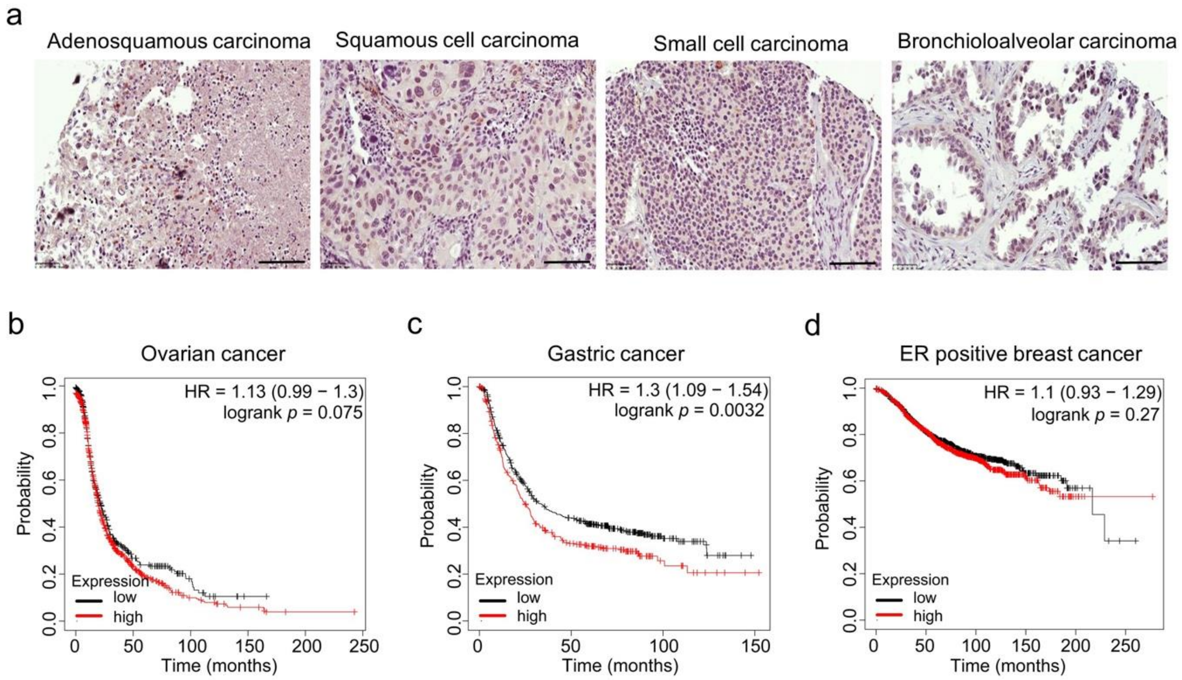

Figure S1. (**a**) IRF9 expression in different types of lung cancer types and its association with survival in various cancer types. IRF9 immunohistochemistry images from adenosquamous carcinoma (n = 7), squamous cell carcinoma (n = 32), small cell carcinoma (n = 6), and bronchioloalveolar carcinoma. (n = 2). Scale bar 100 µm. Kaplan–Meier curves for *IRF9* expression associated with the survival of patients with (**b**) ovarian cancer, (**c**) gastric cancer and (**d**) ER positive breast cancer, divided by the median into high and low expression. Data obtained from Kaplan Meier plotter (Gyorffy et al. 2013).

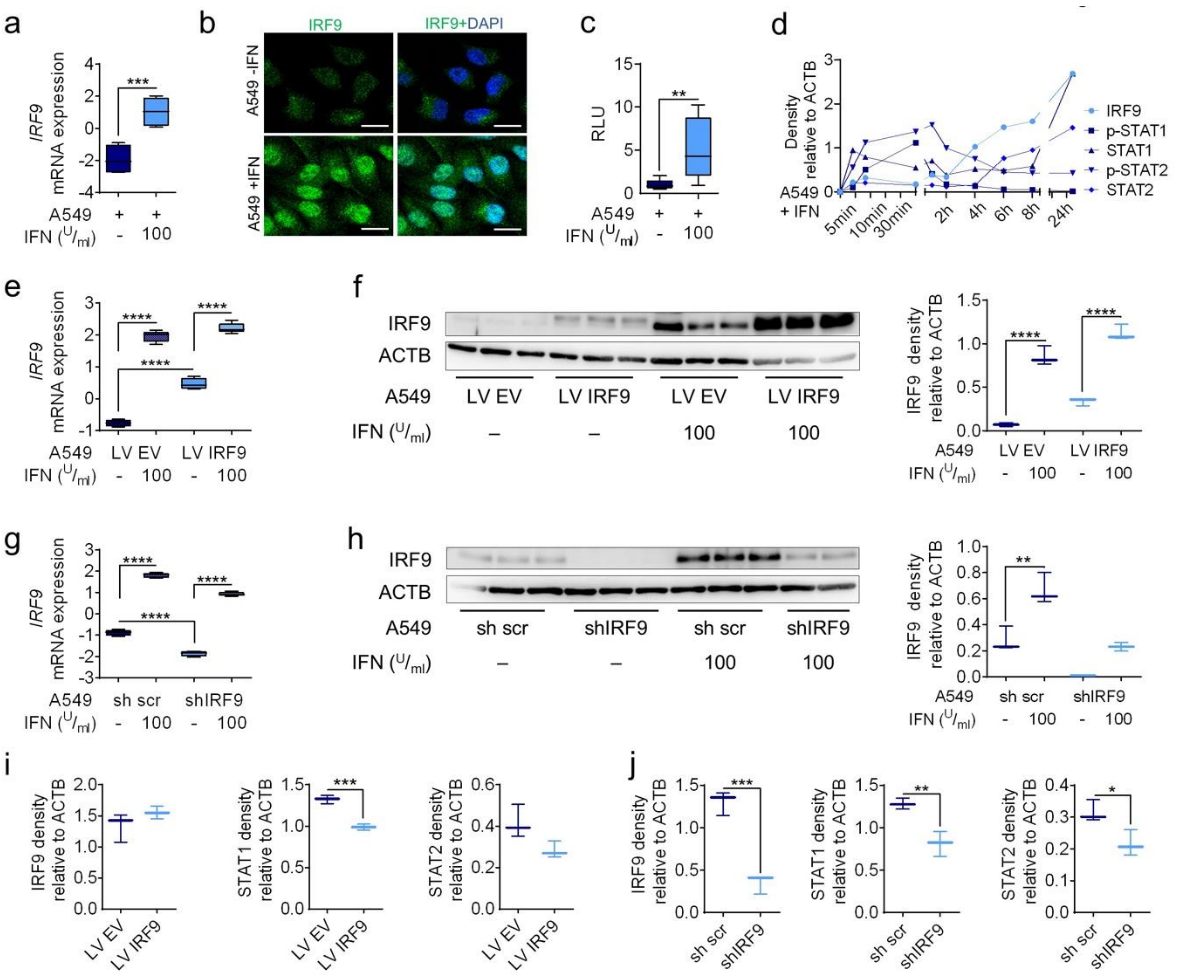

Figure S2: IFN stimulation leads to increased expression and activity of IRF9. A549 cells were stimulated with 100 U/mL Type I IFN for 24 h and expression of IRF9 was evaluated on (**a**) mRNA level and (**b**) cellular level via immunocytochemistry staining using IRF9 antibody and revealed by an AlexaFlour 488 secondary antibody (green). DNA was stained with 4′,6-diamidino-2-phenylindole (Dapi, blue), scale bars, 50 μm (n = 3). (**c**) Activity of IRF9 was measured using a luciferase reporter gene assay with Interferon stimulated response element (ISRE) containing luciferase vector (n = 3). (**d**) Quantification of protein expression via western blot densitometry analysis of IFN stimulated A549 cells at several time points. (**e,g**) Transduced IRF9 tumor cells were stimulated with 100 U/mL Type I IFN for 24 h and expression of *IRF9* was evaluated on mRNA level (n = 2–3) and (**f,h**) protein level, RNA expression was calculated as $\Delta\Delta$Ct. A549 cells treated with IFN compared to A549 without IFN, LV IRF9 compared to LV EV and shIRF9 to sh scr respectively. (**i,j**) Protein expression of IRF9, STAT1 and STAT2 genes in transduced cell lines were quantified via western blot analysis measuring densitometry compared to loading control ACTB (n = 3). Data are shown as mean ± standard error of the mean using Student's t-test or one-way ANOVA. p-values ≤ 0.05 were considered statistically significant for all analyses. * $p < 0.05$; ** $p < 0.01$; *** $p < 0.001$; **** $p < 0.0001$.

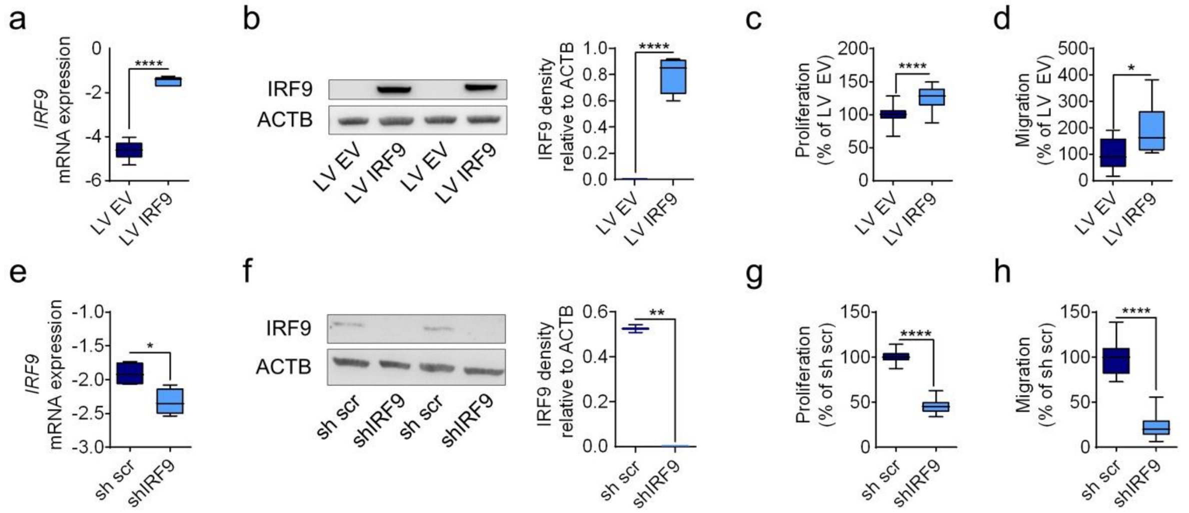

Figure S3: IRF9 transduced A427 cells reveals oncogenic phenotype. (**a,e**) A427 cells were transduced to overexpress IRF9 (LV IRF9) or silence (shIRF9) validated by qRT-PCR. Empty vector (LV EV) or scrambled sequence (sh scr) were used as controls respectively. Western Blot analysis confirmed the (**b**) overexpression or (**f**) silencing of IRF9 at protein level. (**c,g**) Proliferation and (**d,h**) migration of transduced A427 were evaluated as percentage of control respectively. Data are shown as mean ± standard error of the mean using Student's *t*-test. * $p < 0.05$; ** $p < 0.01$; **** $p < 0.0001$; were considered statistically significant for all analyses ($n = 3$).

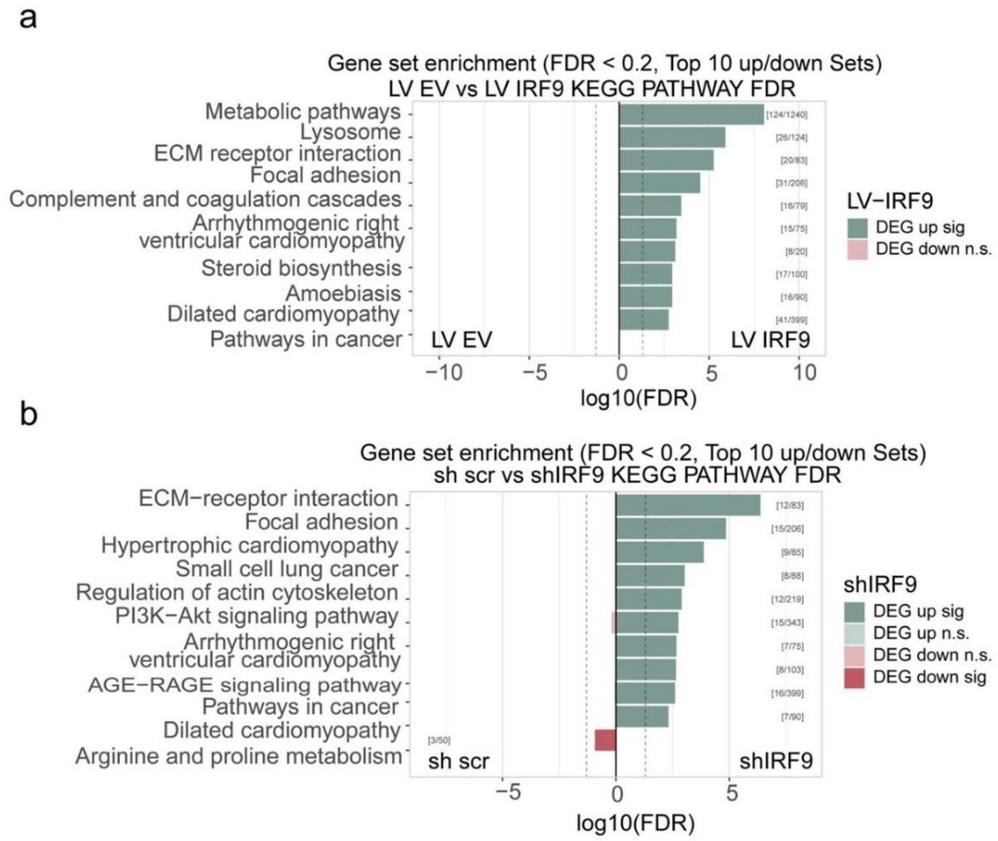

Figure S4: Gene set enrichment analysis of IRF9 overexpressing and silenced A549 cells. (**a**,**b**) Gene set enrichment analysis of KEGG pathway genes in LV IRF9 and shIRF9 transduced A549 cells compared to control vector transduced A549 cells.

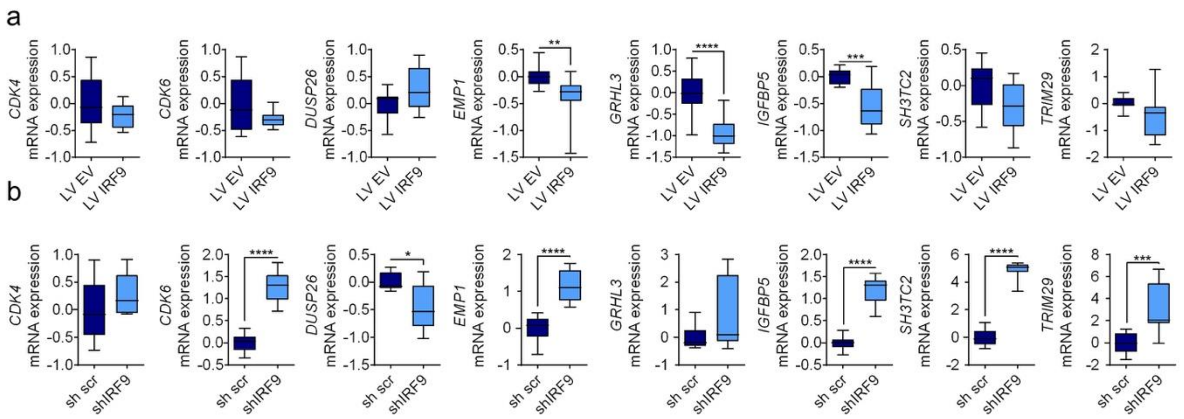

Figure S5: Validation of RNA seq target genes in transduced A549 cells. mRNA expression of potential target genes (*CDK4, CDK6, DUSP26, EMP1, GRHL3, IGFBP5, SH3TC2, TRIM29*) from RNA-seq were validated using qRT-PCR in IRF9 (**a**) overexpressing and (**b**) knockdown cells, calculated as ΔΔCt (n = 2–3). LV IRF9 compared to LV EV and shIRF9 to sh scr respectively. Data are shown as mean ± standard error of the mean using Student's t-test. * $p < 0.05$; ** $p < 0.01$; *** $p < 0.001$; **** $p < 0.0001$; were considered statistically significant for all analyses.

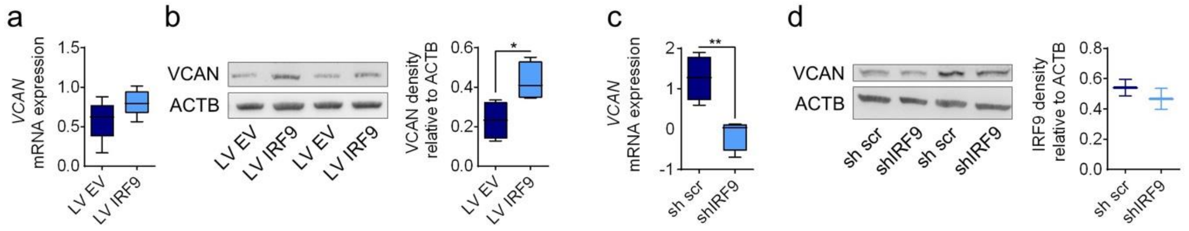

Figure S6: VCAN expression in IRF9 transduced A427 lung cancer cells. Expression of VCAN was evaluated on (**a,c**) mRNA level and (**b,d**) protein level in each cell set of IRF9 transduced A427. LV IRF9 compared to LV EV and shIRF9 to sh scr respectively. Data are shown as mean ± standard error of the mean using Student's t-test. * $p < 0.05$; ** $p < 0.01$; were considered statistically significant for all analyses (n = 3).

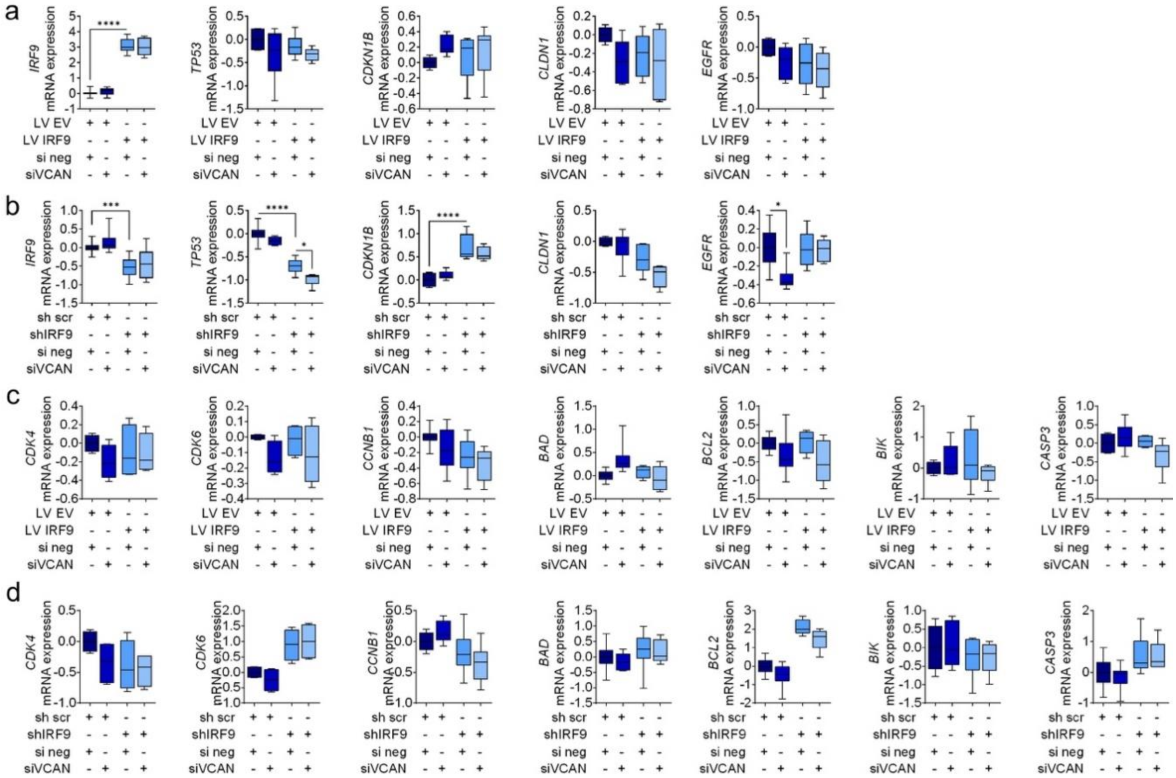

Figure S7: Knockdown of VCAN does not alter genes that are involved in EGF, cell cycle and apoptosis pathways. IRF9 transduced A549 cells were transfected with siRNA against VCAN (siVCAN) to knockdown *VCAN* expression. A non-targeting sequence was used as transfection control (si neg). After knockdown, the expression of indicated genes (*TP53*, *CDKN1B*, *CLDN1*, *EGFR*, *CDK4*, *CDK6*. *CCNB1*, *BAD*, *BCL2*, *BIK* and *CASP3*) was evaluated via qRT-PCR, in (**a,c**) overexpressing A549 and (**b,d**) knockdown A549 compared to A549 LV EV si neg or A549 sh scr si neg respectively. mRNA expression was calculated as ΔΔCt. Data shown as mean ± standard error of the mean using one-way analysis of variance. * $p < 0.05$; *** $p < 0.001$; **** $p < 0.0001$; were considered statistically significant for all analyses ($n = 3$).

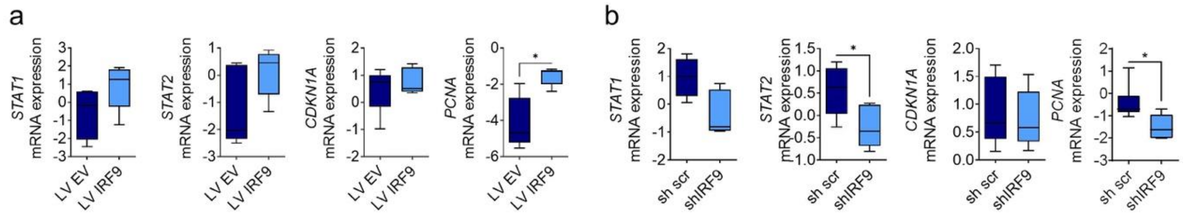

Figure S8: Evaluation of IRF9 associated genes in IRF9 overexpressed or silenced tumors. mRNA expression of *STAT1*, *STAT2*, *CDKN1A* and *PCNA* from tumor homogenate of IRF9 overexpressing (**a**) and silenced (**b**) tumors. Data are shown as mean ± standard error of the mean using Student's *t*-test. * $p < 0.05$; were considered statistically significant for all analyses ($n = 5$).

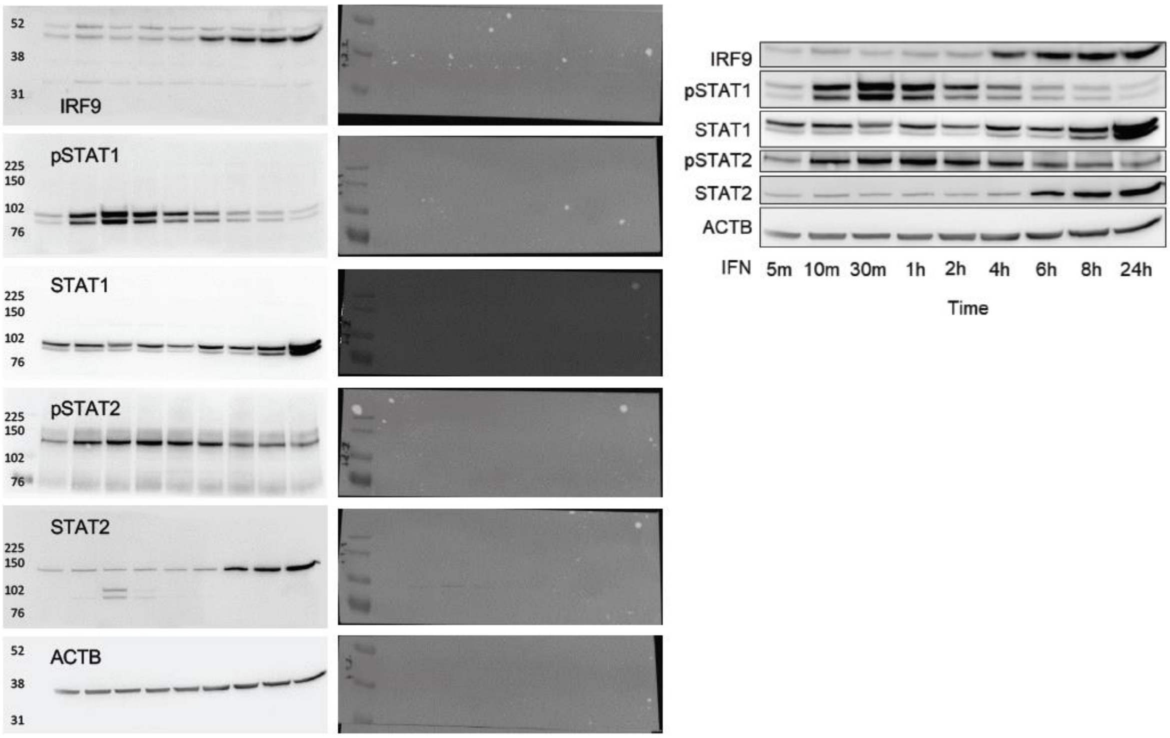

Detail information about Figure 2a.

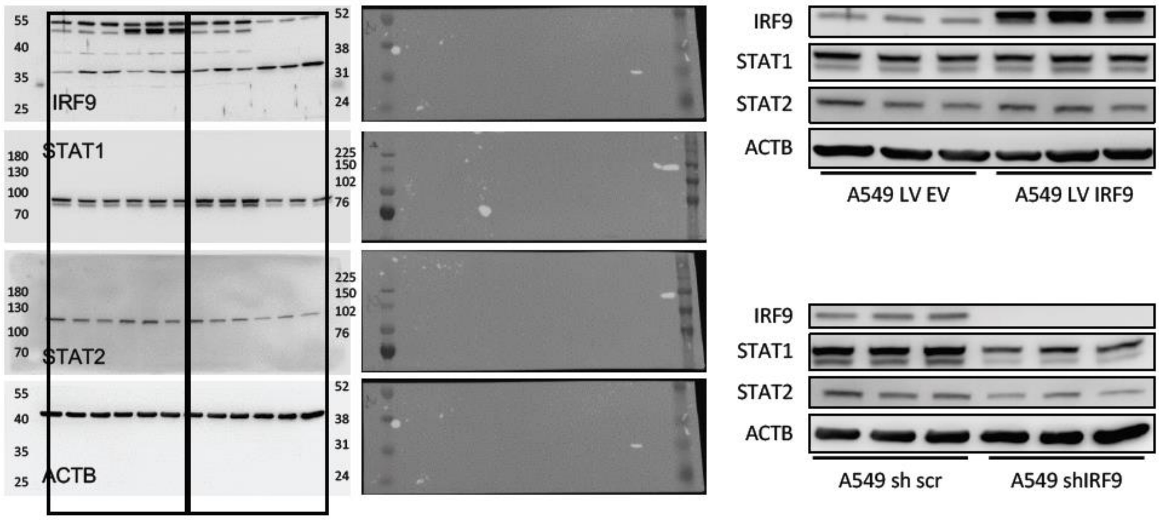

Detail information about Figure 3b,h.

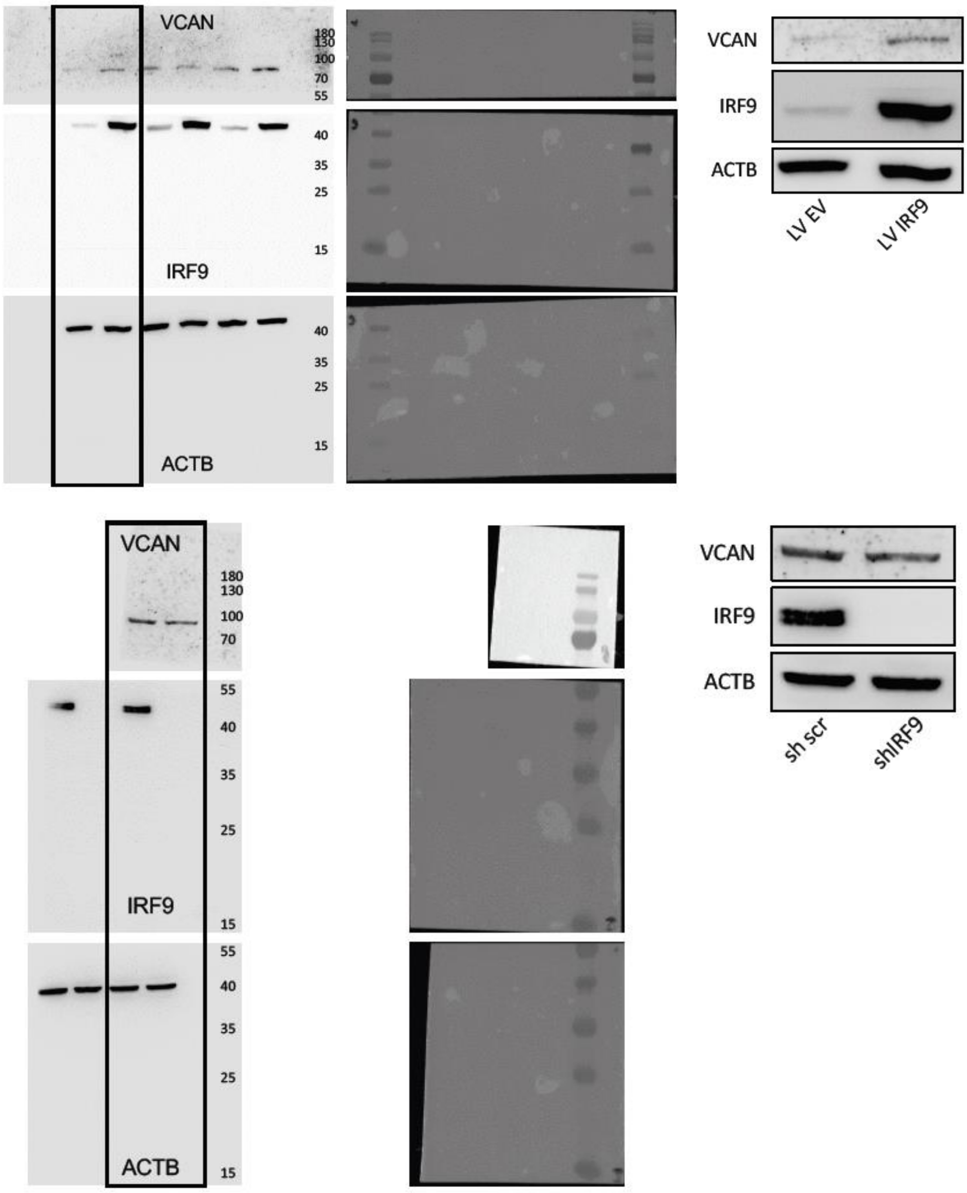

Detail information about Figure 4h,j.

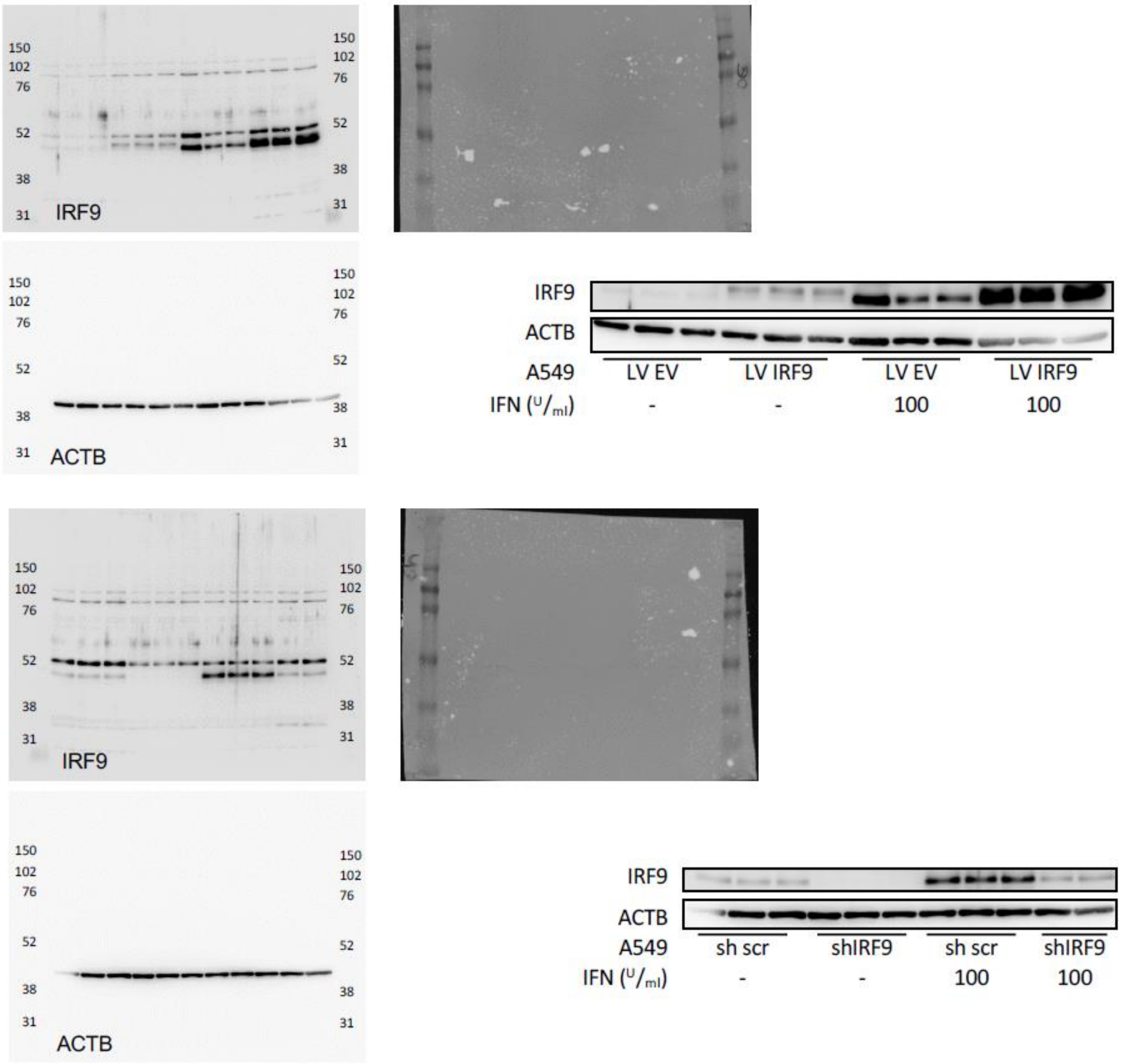

Detail information about Figure S2f,h.

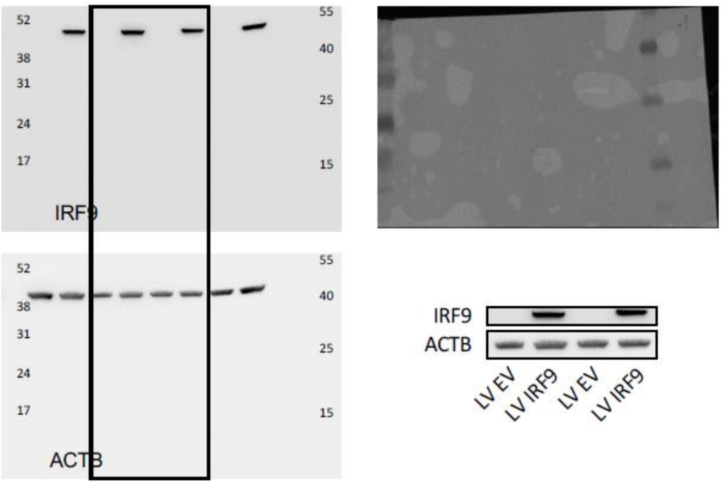

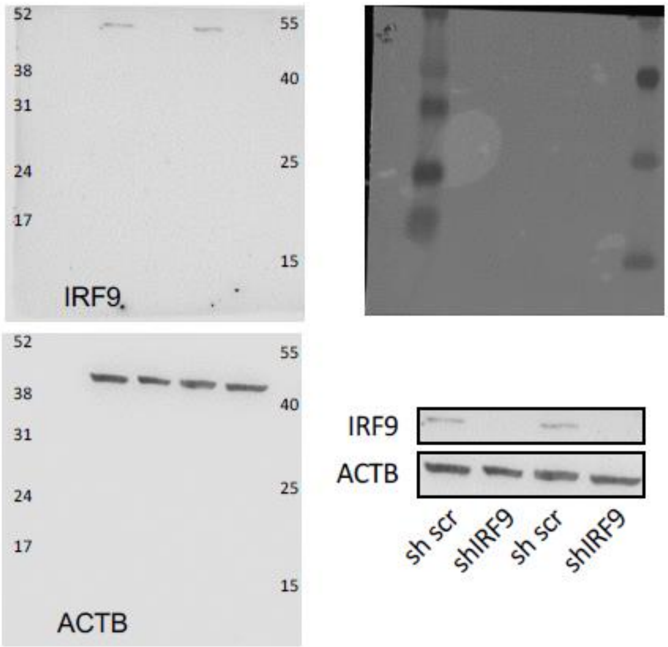

Detail information about Figure S3b,f.

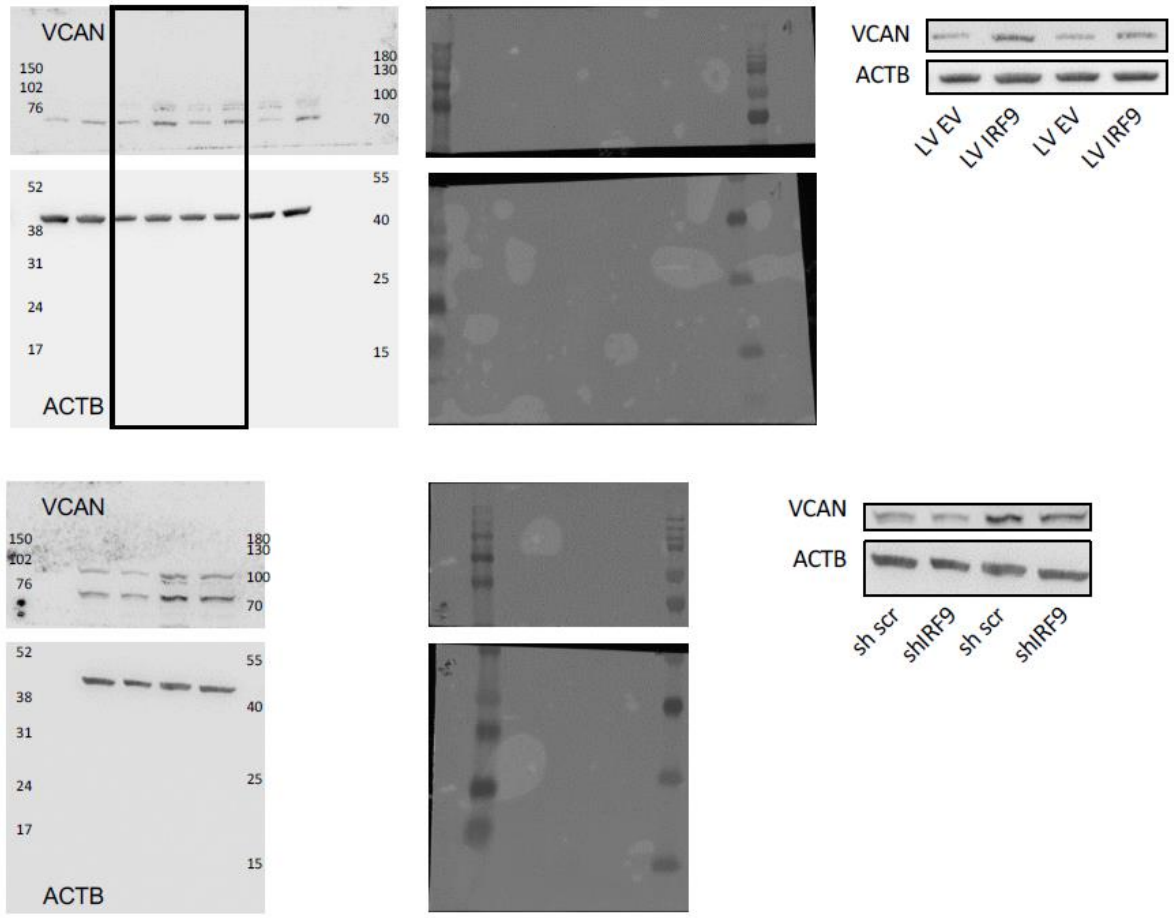

Detail information about Figure S6b,d.

© 2020 by the authors. Submitted for possible open access publication under the terms and conditions of the Creative Commons Attribution (CC BY) license (http://creativecommons.org/licenses/by/4.0/).
